# Understanding How Multiple Strengths Support the Mental Health of Adolescents and Young Adults in a Gender-Diverse Sample

**DOI:** 10.3390/bs16101896

**Published:** 2026-10-14

**Authors:** Victoria Banyard, Kimberly J. Mitchell

**Affiliations:** 1 School of Social Work, Rutgers University, New Brunswick, NJ 08901, USA; 2 Crimes Against Children Research Center, University of New Hampshire, Durham, NH 03824, USA; kimberly.mitchell@unh.edu

**Keywords:** resilience, strengths, mental health, adolescents, young adults

## Abstract

Adolescents and young adults are facing a mental health crisis, with disproportionate burden among youth with diverse gender and sexual identities. Resilience science highlights the importance of modifiable strengths and protective factors as targets for prevention. The Resilience Portfolio Model (RPM) conceptualizes resilience as a multidimensional system of strengths across regulatory, interpersonal, meaning-making, and environmental domains that promote well-being following adversity. This secondary analysis study examined whether strengths across these domains were prospectively associated with mental health and whether these associations differed across social identity groups. Data were drawn from a subsample (N = 1584) from Project Lift Up, a national longitudinal study of 4981 adolescents and young adults (ages 13–22 years), including an oversample of sexual and gender minority youth. Participants completed a baseline survey and follow-ups at six-month intervals. Strengths across the four RPM domains were assessed at different waves (Waves 1–4), and mental health symptoms were assessed at Wave 1 and Wave 5 using the DSM-5-TR Cross-Cutting Symptom Measure. Multiple strengths were independently associated with subsequent global mental health symptoms after adjustment for baseline symptoms, adversity, demographic characteristics, and social determinants of health. Mentoring, hope, self-compassion, and recovering positive affect were associated with lower Wave 5 global symptoms, whereas self-care was associated with higher symptoms. Other interpersonal and environmental strengths were not independently associated with global symptoms. Evidence for moderation was limited to two exploratory interactions involving sexual minority identity, indicating that recovering positive affect and activism may operate differently for these youth. Findings highlight the value of examining multiple dimensions of resilience simultaneously and suggest that regulatory, meaning-making, and selected interpersonal strengths may be particularly relevant to subsequent mental health during adolescence and emerging adulthood.

## 1. Introduction

Recent research documents that adolescents and young adults are experiencing a mental health crisis (McGorry et al., 2025). These concerns are linked to exposure to a variety of adversities including interpersonal violence victimization, adverse childhood experiences, discrimination, exposure to anxiety about how their peers are doing, as well as community and societal level problems and questions about the impact of technology (Abbasi, 2023). These concerns are not equally distributed, with research finding higher rates of psychological distress among communities that differ on sexual identity, gender, race or ethnicity (Depa et al., 2022; Prichett et al., 2024; Watson et al., 2024). Resilience science reminds us of the importance of strengths and protective factors as these may be malleable variables for prevention and intervention/healing efforts to promote well-being among young people (Yoon et al., 2025). Resilience is currently understood as a process by which individuals harness assets and resources to achieve aspects of a good life after exposure to significant adversity or trauma (Banyard et al., 2025a; Hamby et al., 2018a). Importantly, current models are multidimensional, recognizing that resilience is promoted through multiple interacting strengths rather than any single protective factor (Verlenden, 2024). These strengths can be both within the individual (such as their ability to manage strong emotions) but also in their connections to others and resources in their environments. These models allow for individual or group differences in how specific strengths work (or even which strengths are most likely in a group’s toolkit) and move away from resilience as a stable personality trait or a focus on only one particular strength. The current study examined the role of a multidimensional set of strengths in reducing a composite of mental health symptoms prospectively using a national longitudinal sample of adolescents and young adults.

### 1.1. The Resilience Portfolio Model

The current study used one of these multidimensional models, the Resilience Portfolio Model (RPM) (Banyard et al., 2025a). This model organizes strengths into four domains: regulatory (the ability to manage strong feelings and manage routines of sleep and other health behaviors and can be measured as emotion regulation or endurance), interpersonal (positive connections to others as well as skills like communication that support healthy relationships), meaning-making (such as purpose in life and activities that get people outside themselves) and environment (aspects of policies and built and natural spaces). This model has been applied to understanding mental health and indicators of well-being in children, adolescents and young adults (Hébert & Dassylva, 2026; Sabina et al., 2025; Yoon et al., 2024, 2025). A central feature of this model is that strengths measured are modifiable and potential candidates for prevention and intervention strategies rather than measuring resilience as a trait. A core assumption is that resilience emerges from combinations of strengths, these combinations may differ across individuals and groups but also that among these combinations, some strengths may have greater salience to promote well-being than others. To date, studies that include an array of modifiable strengths in their analyses are still fewer in number than studies that center one strength or that measure a composite index of what is termed “resilience.” Studies of the RPM specifically are most often cross-sectional. The current study assessed modifiable strengths in each of the four RPM domains and their relationship to mental health symptoms over time. Furthermore, the current study examined how social identity locations may moderate relationships between strengths and outcomes.

Research supports specific strengths in each of the four RPM domains. Here we summarize evidence for factors that are specific to the current study. Regulatory strengths are those that help individuals manage strong emotions and stress responses including the ability to recover positive emotions after stress, performing self-care activities and routines that can help promote sleep and other regulatory functions, and self-compassion (Egan et al., 2022; Hamby et al., 2020a; Paré-Roy et al., 2025). Interpersonal strengths supported in previous work included mentoring (Hagler et al., 2023), group connectedness and social support (Moisan et al., 2019). Meaning-making includes variables like purpose (Hamby et al., 2020a; Hébert & Dassylva, 2026) but also activism (although research suggests potential mixed relationships here) (Conner et al., 2023a, 2023b) and hope (Assari et al., 2024). Finally, recent attention has been directed at environmental strengths including the built and physical environment, as well as access to natural green spaces with more consistent findings linking nature to well-being and remaining questions about associations with the built environment (Banyard et al., 2025b). The current study included measures of regulatory, interpersonal, meaning-making and environmental strengths.

### 1.2. Resilience Across Diverse Youth

An understudied aspect of the RPM and resilience in general is whether portfolios of strength differ by group such as by aspects of social identity. Most studies of the RPM to date looked at samples as a whole rather than moderating group variables. However, a number of recent scoping reviews on communities that differed by geography or demographics from the more prevalent studies of resilience (centered on North America and predominantly white samples) suggested group differences in profiles of strengths and access to specific strengths (Hagler et al., 2025; Sabina et al., 2025).

There is preliminary evidence that individual strengths may operate differently across groups. For example, Mitchell and colleagues found that while social support was related to lower distress among cisgender young people exposed to suicide thoughts and behaviors in their social networks, social support had the reverse effect and was related to greater depressive symptoms among transgender youth (Mitchell et al., 2021). This finding fits with theories of diminishing returns that posit that communities that experience a disproportionate burden of adversity through systemic and historic oppression and discrimination may experience reduced benefits from some protective factors (Assari, 2017, 2019).

Empirical studies directly examining moderation within the RPM remain limited. For example, Pare-Roy and colleagues’ cross-sectional study of 14–25-year-olds measured strengths across three of the RPM domains. They found differences when comparing three groups (bisexual, gay, heterosexual) on self-reported levels of strengths. Higher sense of purpose, mattering, endurance, and community support were found among heterosexual young people compared to the other two groups. Heterosexual youth and young adults also had higher scores on group connectedness and recovering positive affect and religious meaning-making but only compared to bisexual youth. Within group analyses were conducted to examine strengths related to well-being outcomes. Purpose, positive affect and mattering were related to greater well-being among gay participants. This was similar among bisexual participants for whom teacher engagement and receiving social support were also related to positive outcomes. The analysis for heterosexual youth showed that all of these variables were significant, in addition to social support seeking and endurance (Paré-Roy et al., 2025). Another study (Waterhouse et al., 2025) used cross-sectional data from one wave of a longitudinal study with similar study aims—to look at levels of strength by sexuality and gender and whether aspects of the 5Cs (competence, confidence, character, connection, and caring) of positive youth development frameworks differed across groups among a national sample of 17-year-olds in the United Kingdom. Findings showed high levels of strengths overall. Sexual or gender minority participants had higher odds of reporting a high level of academic qualifications, self-reporting creativity/innovation, volunteering to help others, and kindness but less likely to report having a close friend or feeling close to others including at least one parent. There were also many competency items that did not show significant group differences. More research is needed. The current study enhances this work through the use of longitudinal data and examining moderating associations of social identity and assets with outcomes rather than just group differences in levels of strengths.

Specifically, the current study contained oversampling of young people from communities across gender and sexual identity spectrums. This presented an opportunity to examine variation in the portfolios of a diverse group of adolescents and young adults and to conduct moderation analyses to examine not only whether certain strengths were strongly associated with subsequent mental health concerns as an outcome, but whether some strengths operated differently for subgroups of young people.

### 1.3. Current Study

The current study extended prior RPM research by examining whether a multidimensional portfolio of strengths was associated with mental health beyond demographics, adversity, and baseline symptoms. This study addressed an important gap in research on the RPM by using a longitudinal design for the outcome variable of interest—mental health symptoms. We also looked at how sexual and gender identity moderated the link between strengths and mental health. This adds to the growing understanding of multidimensional resilience by measuring multiple strengths that are potentially modifiable for prevention. Specific aims and hypotheses included the following:

RQ1. To what extent are individual strengths assessed across Waves 1–4 prospectively associated with Wave 5 mental health symptom severity, above and beyond baseline symptoms, adversity, and social determinants of health? Based on previous research, we hypothesized that while all strengths would be related to lower symptoms at the bivariate level, only a few would be significant in multivariate models. We hypothesized that strengths from each RPM domain would demonstrate prospective associations with lower symptom burden.

RQ2. How do associations between strengths and Wave 5 symptom severity differ by sex assigned at birth, gender identity, and sexual identity? Previous research did not suggest specific patterns of moderation here and this aim was exploratory.

RQ3. Which strengths assessed across Waves 1–4 are most consistently associated with lower subsequent symptom burden across mental health domains? Previous research suggested that different strengths were more significantly related to different well-being outcomes in some groups than others (especially purpose, receiving social support, and emotion regulation) but less is known about differences among groups of mental health symptoms. This was also an exploratory aim.

## 2. Materials and Methods

### 2.1. Study Population

Project Lift Up is a longitudinal, national study of youth and young adults designed to understand bystander behaviors for self-directed violence. The sample of 4981 adolescents and young adults aged 13–22 years was recruited between 13 June 2022 and 30 October 2023. Four additional waves of data were collected, approximately 6 months apart: Wave 2 (collected between 13 January 2023–26 February 2024); Wave 3 (13 June 2023–19 November 2024); Wave 4 (15 December 2023–8 May 2025), and Wave 5 (12 June 2024–21 October 2025). Although the calendar periods for later study waves partially overlapped because of rolling participant enrollment, each participant completed assessments sequentially; thus, all strength measures preceded that participant’s 24-month (Wave 5) mental health assessment. The current analyses represented secondary analyses as they used variables that were not part of the primary funded aims of the study.

Of the 6237 baseline surveys originally submitted, 4981 (79.9%) were deemed valid and retained for analysis. The remaining responses were excluded due to failure to meet age requirements (9.3%), evidence of large-scale duplicate or fraudulent submissions from outside the United States (4.1%), or smaller-scale duplicate entries (6.7%). Further information on these validation procedures is available elsewhere (Mitchell et al., 2025a, 2025b). The qualifying longitudinal cohort, defined as completing baseline and either the Wave 2 or Wave 3 surveys, was 3360. The final complete-case analytic sample included 1584 participants (47.1% of the qualifying cohort) with complete data on all key constructs required for the primary analyses. Comparisons between participants included in the final analytic sample and those excluded because of missing data on key constructs are presented in Table 1. Overall, the two groups were similar with respect to age, sexual minority identity, Hispanic ethnicity, and most baseline social determinants of health. Participants included in the analytic sample were more likely to be female, less likely to identify as a gender minority, less likely to be white, and more likely to be Asian, and reported slightly lower baseline global symptom severity and financial instability than excluded participants.

### 2.2. Procedures

Participants were recruited through study advertisements on social media, including Facebook and Instagram, which linked them to a secure survey website. Those who were eligible (i.e., 13–22 years of age, living in the United States, proficient in English), were then asked to read an assent/consent form and to indicate their willingness to participate in the survey before continuing with the main survey. Ineligible youth were directed to a web page that included links to general resources for youth (e.g., https://youngwomenshealth.org, accessed on 1 March 2022). Participants were given a $15 incentive as an Amazon gift code for completing Waves 1–3 (Wave 3 also had a $5 early completer bonus if they finished the survey in the first 24 h after receiving their invitation), $20 for completing Wave 4, and $25 at Wave 5 (with a $5 early completer bonus).

To promote a diverse sample with sufficient sample size of young people from communities disproportionately affected by various mental health outcomes, especially suicide exposure, demographic quotas were identified. Once the targeted number of youth in a particular group had been achieved (e.g., aged 13–17, cisgender girls), subsequent youth in this group who were otherwise eligible were deemed ineligible.

The protocol was reviewed and approved by the University of New Hampshire Institutional Review Board. A waiver of caregiver permission was requested and granted by the IRB for minors because requiring caregiver consent could potentially place youth in situations where their sexual experiences and/or sexual attraction could be unintentionally disclosed to their parents given this study oversampled less prevalent sexual and gender identity groups. A multi-stage data validation procedure was implemented at baseline to detect and remove invalid or unreliable responses. This protocol involved confirming age eligibility, conducting reverse checks on IP addresses and phone numbers, reviewing contact details and social media handles, evaluating survey completion times, incorporating attention-check questions, and following up with respondents when needed.

### 2.3. Measures

#### 2.3.1. Strengths

A suite of strength-based measures was included across different waves of this study to assess protective psychosocial resources using the RPM. Measures are described within domains of the RPM where they are typically studied. To standardize each strength, z-scores were calculated for all continuous scales to allow for the comparison of values. Because strengths and resilience were not the primary funded aims of Project Lift Up, measures of strengths were incorporated across multiple survey waves as survey space permitted. Consequently, strengths were assessed at different study waves and were not measured repeatedly over time.

##### Regulatory Domain

Emotional regulation, captured at Wave 3, was measured with the Recovery of Positive Affect scale (Hamby et al., 2020a), a six-item measure assessing participants’ ability to cultivate positive emotional states (e.g., “I can still laugh at a joke, even when I’m having a bad day”). Responses on a four-point scale ranging from 1 (not true about me) to 4 (mostly true about me) provided a measure of psychological flexibility (α = 0.75, Mean = 2.95, SD = 0.6, Range: 1–4).

Self-compassion was measured at Wave 4 with selected items from the Self-Compassion Scale for Youth developed by Neff et al. (2021). This measure included items from the self-kindness subscale in its entirety, one item from the common humanity subscale (with minor wording adjustments for language clarity), and two mindfulness items. A sample item was “I am kind and supportive to myself when I’m having a hard time.” Wording across all items was slightly adapted for accessibility while preserving the original construct definitions. Participants were asked to respond to each statement indicating how often “you think this about yourself when you are having a hard time”; response options ranged from 1 (almost never) to 5 (almost always) (α = 0.87, Mean = 2.90, SD = 0.9, Range: 1–5).

Self-care items were created for the Wave 2 survey and examined things people do to take care of themselves and promote regulation and coping with stress; items were informed by a review of the literature (Town et al., 2024) and current clinical practice guidelines, although much of this work focused on adults and health care professionals or on self-care among people with disabilities (Hansson et al., 2005; Jiang et al., 2021a). Indices for adolescents often focused on positive health-related and developmental rule-following behaviors (Urpí-Fernández et al., 2017; Jiang et al., 2021b). Participants were prompted to think about 14 different things people do to take care of themselves in a typical month, including exercise, creative expression, mindfulness practices, and interactions with pets, with space provided to specify additional strategies they personally used. Response options ranged from 1 (never) to 5 (always). For the present analyses, each item was dichotomized to indicate whether the participant engaged in that activity at least sometimes during a typical month (0 = never; 1 = sometimes, half the time, often, or always). Items were dichotomized because the construct of interest was the breadth of self-care strategies used rather than the frequency of any particular activity. Dichotomized items were summed to create a count of different self-care activities used (possible range = 0–14), with higher scores indicating engagement in a greater variety of self-care strategies (α = 0.65, Mean = 7.87, SD = 2.7, Range: 0–14). The resulting measure was conceptualized as an index of accumulated self-care behaviors rather than a reflective scale; individual behaviors were not necessarily expected to co-occur.

##### Meaning-Making Domain

The measure of activism was from the Critical Consciousness Scale developed by Diemer et al. (2017) and assessed at Wave 2. Minor adaptations included simplifying language (e.g., removing outdated references like “by phone, mail, or email”) to better reflect contemporary forms of youth activism. Items captured youths’ engagement in various social justice activities, such as participation in protests, political organizations, and advocacy efforts, over the past year. Participants were asked to respond as to how often they were involved in each activity in the past year with options ranging from 1 (never did this in the past year) to 5 (about once a week) (α = 0.81, Mean = 1.72, SD = 0.6, Range: 1–5).

Participants’ hope was assessed at Wave 3 using six items adapted from Krafft and colleagues’ validated hope scale (Krafft et al., 2019). This scale captured both dispositional and situational hope by asking participants to rate their agreement with statements such as “I feel hopeful” and “Even in difficult times, I am able to remain hopeful.” Response options ranged from 1 = strongly disagree to 6 = strongly agree. Items were averaged to create a composite hope score, with higher scores indicating greater hope (α = 0.93, M = 3.84, SD = 1.10; observed range = 1–6).

Participants’ sense of purpose (Hamby et al., 2018b) was assessed at Wave 3 using six items such as “My life has a clear sense of purpose” and “My values give my life meaning” that were rated on a 4-point scale from 1 = not true about me to 4 = mostly true about me (α = 0.90, Mean = 2.83, SD = 0.9, Range: 1–4).

##### Interpersonal Domain

Social support was assessed at Wave 4 through two validated scales developed by Hamby et al. (2018b): the Social Support Receipt Scale and the Social Support Seeking Scale. For social support receipt, participants were prompted to think about the last time they were upset about something and then answer five questions about how true each was about them. For example, “Someone went with me to get some help” (α = 0.87, Mean = 1.63, SD = 0.9, Range: 0–3). Social support seeking more generally assessed participants’ propensity to seek emotional or instrumental support from their social networks. It consisted of six items. A sample item was “I feel better when I talk to people about what’s going on” (α = 0.88, Mean = 2.14, SD = 0.8, Range: 0–3). Response options for both scales ranged from 0 (not true about me) to 3 (mostly true about me).

Social connections assessed feelings of closeness and support from peer groups with seven items captured at Wave 4 (Hamby et al., 2020a). Six of the items were modified from “group or team” to “friends.” One item was created for the current study to ask about feeling safe among friends. Response options ranged from 1 (not true about me) to 4 (mostly true about me) (α = 0.84, Mean = 3.4, SD = 0.5, Range: 1–4). An example item was “I belong to a group or team that means a lot to me.”

At Waves 1 and 3, participants were asked if they had received any suicide prevention messaging from different sources: (1) family, (2) friend, (3) website, (4) talk or presentation, (5) specific program, (6) posters or flyers, and (7) social media. The Wave 1 survey asked about lifetime and the Wave 3 survey asked about the past 12 months. A positive response at either wave was coded as having received suicide prevention messaging. Because prevention is interactive, this was considered part of the interpersonal domain of strengths. This measure has been used in previous work (Banyard et al., 2022).

At Wave 2, participants were provided with the following preamble following work by Zimmerman et al. (2002): “This next question asks about having a mentor. By mentor we mean someone who is older than you that you can go to for support and advice, or when you need to make an important decision. A mentor can also be someone who inspires you or shows you how to be your best. Other than your parents, step-parents or close friends, do you have a mentor who makes a positive difference in your life?” Response options were yes or no.

##### Environmental Domain

Access and engagement with positive features of the built and natural environment (community/environment resources) was assessed at Wave 4 using a newly developed item set assessing access to and use of 18 different community resources, including parks, public transportation, libraries, and food pantries. For each resource, participants indicated whether it existed in their area, was accessible, and had been used in the past year (yes/no). This structure was informed by prior work on community assets and environmental supports for youth well-being (Saelens et al., 2003). The current analyses utilized a count of different resources they had used in their community (α = 0.77, Mean = 4.68, SD = 3.3, Range: 0–18). Because this measure captures a heterogeneous set of community resources, use may reflect access, availability, engagement, or need rather than a single underlying environmental construct.

#### 2.3.2. Outcome

Global Cross-Domain Symptom Severity Index: Participants completed the DSM-5-TR Cross-Cutting Symptom Measure (American Psychiatric Association, 2024) at Wave 1 and Wave 5. The measure assesses multiple domains of psychopathology, including somatic complaints, sleep problems, inattention, depression, anger, mania, anxiety, psychosis, repetitive thoughts/behaviors, and substance use (count). Because this measure does not provide a validated overall score, we derived a study-specific global cross-domain symptom severity index to characterize participants’ overall burden of psychopathology across domains. This is consistent with research using the measure as a more global symptom inventory and with theory about a general domain of psychopathology (Caspi et al., 2014; Gibbons et al., 2023; Lace & Merz, 2020) and supported by a Cronbach alpha of 0.90 for the current sample. At each assessment wave, domain scores were standardized (z-scored) within the analytic sample before averaging so that each symptom domain contributed equally to the composite despite differences in score ranges and variances. Higher scores indicate greater overall cross-domain symptom severity. The resulting index was re-standardized (M = 0, SD = 1) to facilitate interpretation in regression models, with higher values reflecting greater overall cross-domain symptom severity relative to the sample mean. Supplemental Files present analyses using the more conventional symptom subscales.

#### 2.3.3. Adversity and Risky Social Determinants of Health

Lifetime adversity was assessed at Wave 1 with 12 items capturing youth and family-level experiences of economic hardship, familial separation, parental incarceration, chronic illness, and substance misuse. These items were adapted from Turner and Butler’s (2003) work on chronic and non-violent adversity. Examples included “Did your mother, father, or guardian lose a job?” and “Did someone you know overdose on drugs and get really sick?” (α = 0.66, Mean = 3.01, SD = 2.2, Range: 0–10).

Social Determinants of Health

Indicators of SDoH-related hardship were assessed using items drawn from multiple sources, including the American Academy of Family Physicians (AAFP) social needs screening tool (American Academy of Family Physicians, 2024), and the Youth Risk Behavior Surveillance System (Centers for Disease Control, 2020). Items were selected for developmental appropriateness and suitability for self-administration, given that many existing SDoH measures are designed for adults or clinical settings. These measures have been utilized in other publications from the same study (Mitchell et al., 2025b) and additional studies by the current authors (Mitchell et al., 2025a).

Financial instability was indicated by assessing difficulty paying bills; food insecurity was queried based on a question about skipping meals due to lack of money (5-point scale from never to always) (American Academy of Family Physicians, 2024). Barriers to mental health care were assessed using five items capturing logistical and financial obstacles (e.g., cost, transportation, scheduling), rated from strongly disagree to strongly agree and summed to create a total score (Britt et al., 2008). Poor home conditions were measured using eight yes/no items assessing housing problems (e.g., mold, pests, inadequate heat, electrical outages), summed to create a total score (American Academy of Family Physicians, 2024). Community disorder was assessed with 12 items capturing perceptions of neighborhood problems (e.g., graffiti, violence, gunshots, abandoned buildings), rated as no problem, minor problem, or serious problem (Perkins et al., 1990). Discrimination was assessed using the Everyday Discrimination Scale (Williams et al., 1997); participants reported the frequency of unfair or demeaning treatment in daily life (e.g., people act as if they are better than you are).

#### 2.3.4. Demographic Characteristics (Wave 1)

Sexual and Gender Identity: To measure sexual identity, participants were asked “which best represents how you think of yourself”: gay; lesbian, bisexual; heterosexual; queer; polysexual, omnisexual, sapiosexual, or pansexual; demisexual; asexual; two-spirit; do not think of myself as having sexuality; do not use labels to identify myself; questioning/I am unsure; and something else. These response options were reduced to a single dichotomous variable of exclusively heterosexual (0) versus all others (designated as sexual minority = 1). Participants were asked what terms best describe their gender identity and were given a list: cisgender boy/man; transgender boy/man; cisgender girl/woman; transgender girl/woman; non-binary; genderqueer; genderfluid; pangender; agender; indigenous or other cultural gender minority identity; questioning or unsure of your gender identity; and something else. This multiple-response measure was reduced to a single dichotomous variable for the current analyses: cisgender (coded as 0) and gender minority (coded as 1).

Race response options included White, Black or African American, Asian, Hawaiian or Pacific Islander, American Indian or Alaska Native, Unknown or not reported, other, and prefer not to answer. Participants were also asked if they were of Hispanic or Latino origin (yes/no). Age was a continuous variable ranging from 13 to 22 years (M = 17.4 (2.2)). The answers for sex assigned at birth were male, female, intersex, and prefer not to answer.

### 2.4. Data Analysis

As noted previously, this was a secondary analysis. All analyses were conducted in StataNow/SE 19.5. Primary aims were to (1) estimate the incremental contribution of youth strengths to Wave 5 mental health outcomes beyond demographics/social determinants of health (SDoH), baseline symptoms, and adversity; and (2) test whether associations between strengths and Wave 5 global symptoms differed by key identity groups (sex assigned at birth, gender identity, and sexual identity) using moderation analyses.

#### 2.4.1. Outcomes

Outcomes were mental health indicators assessed at Wave 5. The primary outcome was global symptom severity, operationalized as a standardized composite (z-scored). Secondary outcomes were domain-specific symptoms (z-scored): somatic symptoms, sleep symptoms, inattention, depression, anger/irritability, mania/hypomania, anxiety, psychosis, repetitive thoughts/behaviors, and substance use count (standardized).

#### 2.4.2. Key Constructs and Block Structure

Hierarchical (nested) linear regression models were estimated using Stata’s nestreg command. Constructs were entered in three sequential blocks to evaluate incremental explained variance and consistent with previous work on the RPM (Hamby et al., 2018a): (1) Block 1—Demographics and SDoH; (2) Block 2—Wave 1 mental health symptoms and adversity (for the global outcome, baseline global symptom severity was entered; for domain-specific models, the corresponding baseline domain score was entered; and (3) Block 3—Strengths. Strengths assessed across Waves 1–4 were entered together in Block 3 because the analytic aim was to estimate their independent prospective associations with Wave 5 symptoms within the multidimensional resilience portfolio. All strength assessments preceded each participant’s Wave 5 outcome assessment. Because individual strengths were assessed at specific waves rather than repeatedly at each wave, measurement timing could not be separated statistically from the strength construct itself. Model improvement was evaluated using ΔR^2^ and F-change tests for each added block. Unstandardized coefficients (B) with standard errors (SEs) and *p*-values are reported for main models. For domain outcomes, both unstandardized (B) and standardized coefficients (β) from the final (Block 3) models were reported. Variance inflation factors (VIFs) were examined for the fully adjusted model to assess multicollinearity among predictors.

#### 2.4.3. Moderation Analyses

Moderation analyses focused on Wave 5 global symptom severity. Three separate moderation models were estimated, each adding a fourth block of interaction terms between each strength and one moderator: (1) Sex assigned at birth, (2) Gender identity; and (3) Sexual identity. Interaction terms were entered simultaneously in Block 4 after Blocks 1–3. Binary strengths were specified using factor-variable notation, and continuous strengths were interacted using Stata’s continuous prefix to ensure correct product-term estimation. Because the moderation analyses involved multiple interaction tests, *p* values for individual interaction terms were adjusted using the Benjamini–Hochberg false discovery rate (FDR) procedure. Only interactions that remained statistically significant following FDR correction were interpreted. Significant interactions were probed using estimated marginal effects (simple slopes; dy/dx) within moderator groups, and predicted values were visualized using margins plots. As a sensitivity analysis, the more conservative Bonferroni correction was also applied and yielded identical substantive conclusions. For RQ3, the Benjamini–Hochberg FDR procedure was also applied across the 120 strength-by-domain tests (12 strengths × 10 symptom domains). Domain-specific associations were considered statistically significant after multiple-testing correction at an FDR-adjusted *p* < 0.05.

#### 2.4.4. Estimation, Inference, and Missing Data

All models were estimated using ordinary least squares regression. Tests were two-tailed with α = 0.05. For the primary regression analyses, complete-case estimation was used, requiring complete data on all constructs included in Blocks 1–3 and complete Wave 5 outcome data. This resulted in a final analytic sample of N = 1584, which is used for all primary and supplemental regression analyses. To evaluate the robustness of the primary findings to differential inclusion in the complete-case analytic sample, we conducted an inverse-probability-weighted sensitivity analysis. The probability of inclusion in the analytic sample was estimated among the qualifying longitudinal cohort (N = 3360) using logistic regression. The model included baseline age, sex assigned at birth, sexual minority identity, gender minority identity, race/ethnicity, food insecurity, financial instability, mental health care barriers, residential instability, poor home conditions, discrimination, global mental health symptoms, and adversity. Predicted probabilities from this model were used to construct stabilized inverse-probability-of-inclusion weights using the marginal probability of inclusion as the numerator. The fully adjusted primary model was then re-estimated using these weights, and the weight distribution was examined for extreme values.

## 3. Results

### 3.1. Bivariate Relationships Between Individual Strengths, Mental Health, and Adversity

Overall, individual strengths were significantly correlated with each other as well as global mental health and adversity (Table 2). Most strengths were negatively correlated with mental health and adversity. A few exceptions were noted: activism and self-care were both associated with greater self-reported global mental health symptoms and greater adversity for the full sample.

### 3.2. Hierarchical Regression Examining Wave 5 Global Symptom Severity

A nested linear regression examined correlates of Wave 5 global symptom severity (Table 3). Constructs were entered in three blocks.

#### 3.2.1. Block 1: Demographics and SDoH

Block 1 explained 17.5% of the variance in Wave 5 global symptoms (R^2^ = 0.175; F(14, 1569) = 23.73, *p* < 0.001; Table 3). Higher symptom severity was associated with female sex at birth (B = 0.116, *p* = 0.015), sexual minority identity (B = 0.169, *p* = 0.002), gender minority identity (B = 0.259, *p* < 0.001), food insecurity (B = 0.084, *p* = 0.001), mental health care barriers (B = 0.115, *p* < 0.001), poor home conditions (B = 0.051, *p* = 0.044), and discrimination exposure (B = 0.164, *p* < 0.001). Other demographic covariates were not significant at this stage.

#### 3.2.2. Block 2: Baseline Symptoms and Adversity

Adding baseline global symptoms and adversity significantly improved fit (ΔR^2^ = 0.170; F-change (2, 1567) = 203.50, *p* < 0.001), yielding R^2^ = 0.345. Wave 1 global symptoms were strongly associated with Wave 5 symptoms (B = 0.463, *p* < 0.001), and adversity independently was associated with higher Wave 5 symptoms (B = 0.078, *p* = 0.002). After adjustment for Wave 1 symptoms/adversity, most Block 1 effects attenuated; gender minority identity remained significant (B = 0.140, *p* = 0.009).

#### 3.2.3. Block 3: Strengths

Adding strengths significantly improved model fit (ΔR^2^ = 0.050; F-change (12, 1555) = 10.77, *p* < 0.001), resulting in R^2^ = 0.395. In the fully adjusted model, Wave 1 global symptoms (B = 0.375, *p* < 0.001) and adversity (B = 0.068, *p* = 0.005) remained robust correlates. Several strengths were independently associated with lower Wave 5 global symptoms, including mentoring (B = −0.120, *p* = 0.004), hope (B = −0.159, *p* < 0.001), self-compassion (B = −0.098, *p* < 0.001), and recovering positive affect (B = −0.048, *p* = 0.047). In contrast, self-care activities were associated with higher Wave 5 symptoms (B = 0.087, *p* < 0.001). Other strengths were not statistically significant. Variance inflation factors indicated little evidence of problematic multicollinearity (mean VIF = 1.38; maximum VIF = 2.65 for hope; VIF for purpose = 2.50).

Results were substantively similar in the inverse-probability-weighted sensitivity analysis (Supplemental Table S1). Stabilized weights were well behaved (mean = 1.00; 99th percentile = 1.47; maximum = 1.96). Mentoring (B = −0.118, 95% CI [−0.200, −0.036], *p* = 0.005), hope (B = −0.163, 95% CI [−0.235, −0.091], *p* < 0.001), self-compassion (B = −0.094, 95% CI [−0.147, −0.041], *p* = 0.001), and recovering positive affect (B = −0.059, 95% CI [−0.111, −0.007], *p* = 0.027) remained associated with lower Wave 5 global symptoms, while self-care remained associated with higher symptoms (B = 0.081, 95% CI [0.034, 0.129], *p* = 0.001). Social connectedness was also associated with higher symptoms in the weighted model (B = 0.043, 95% CI [0.002, 0.083], *p* = 0.038), although its estimate was similar in magnitude to the primary complete-case model. Overall, the pattern of findings was similar to the primary analysis, providing evidence of robustness to differential inclusion associated with the measured baseline characteristics incorporated into the weighting model.

### 3.3. Strengths Across Domain-Specific Wave 5 Outcomes

Block 3 associations between strengths and each standardized Wave 5 domain outcome are summarized in Table 4, with full model results (including B, SE, β, raw and FDR-adjusted *p* values) reported in Supplemental Tables S2–S11. Benjamini–Hochberg FDR correction was applied across the 120 strength-by-domain tests (12 strengths × 10 symptom domains); 24 associations remained statistically significant after correction. Self-care activities were positively associated with somatic, sleep, inattention, depression, anger/irritability, and anxiety symptoms. Hope was inversely associated with somatic, sleep, depression, anxiety, psychosis, and repetitive thoughts/behaviors. Self-compassion was inversely associated with somatic, inattention, depression, anger/irritability, and anxiety symptoms. Mentoring was inversely associated with somatic, inattention, and anxiety symptoms, and recovering positive affect was inversely associated with depression, anger/irritability, and anxiety symptoms. Community/environment resource use was positively associated with substance-use symptom count. No strength associations with mania/hypomania remained statistically significant after FDR correction.

### 3.4. Moderation of Strengths on Wave 5 Global Symptoms

Because the moderation analyses involved multiple interaction tests, individual interaction *p*-values were adjusted using the Benjamini–Hochberg false discovery rate procedure. Unless otherwise noted, only interactions remaining statistically significant after correction are interpreted below.

#### 3.4.1. Sex Assigned at Birth Moderation

In the sex-at-birth moderation model (Supplemental Table S12), model fit increased across Blocks 1–3 in the same pattern as the primary model (R^2^ = 0.1747, 0.3449, 0.3951). Adding the full set of strength × sex-at-birth interactions in Block 4 did not improve model fit (ΔR^2^ = 0.006; F-change (12, 1543) = 1.23, *p* = 0.256; final R^2^ = 0.401). Following FDR correction, no individual interaction terms remained statistically significant, providing limited evidence that associations between strengths and Wave 5 global symptom severity differed by sex assigned at birth (Table 5).

#### 3.4.2. Gender Identity Moderation

In the gender minority moderation model (Supplemental Table S13), adding the interaction block significantly improved fit (ΔR^2^ = 0.013; F-change (12, 1543) = 2.74, *p* = 0.0011; final R^2^ = 0.4078). However, no individual strength × gender minority identity interaction remained statistically significant following FDR correction. Thus, although the interaction block improved overall model fit, there was insufficient evidence to identify a specific strength whose association with Wave 5 global symptom severity differed by gender minority identity (Table 5).

#### 3.4.3. Sexual Identity Moderation

**Figure 1:**
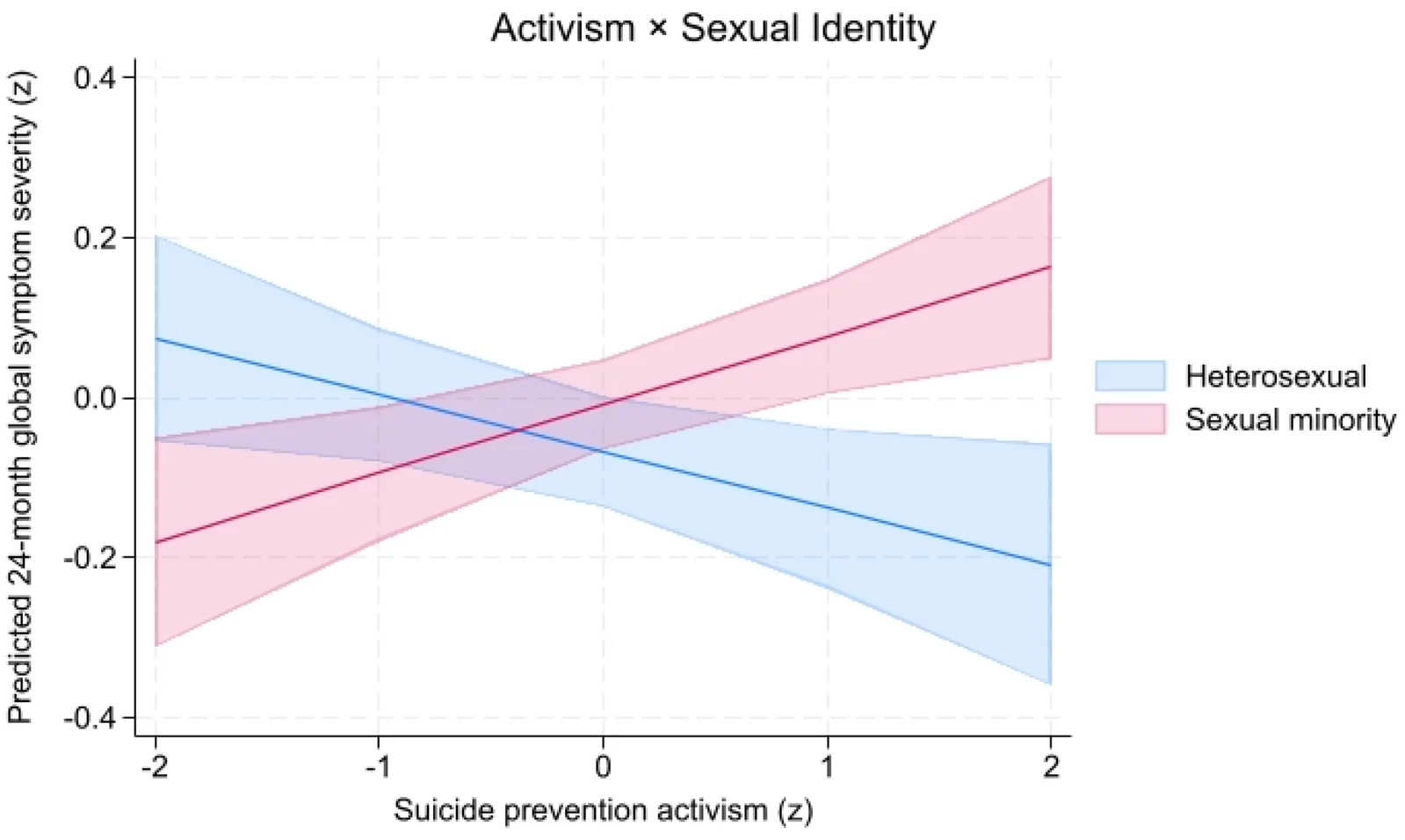
Interaction between activism and sexual identity on Wave 5 global symptom severity. Predicted standardized Wave 5 global symptom severity across levels of suicide prevention activism by sexual identity from the fully adjusted moderation model. Shaded bands represent 95% confidence intervals.

**Figure 2:**
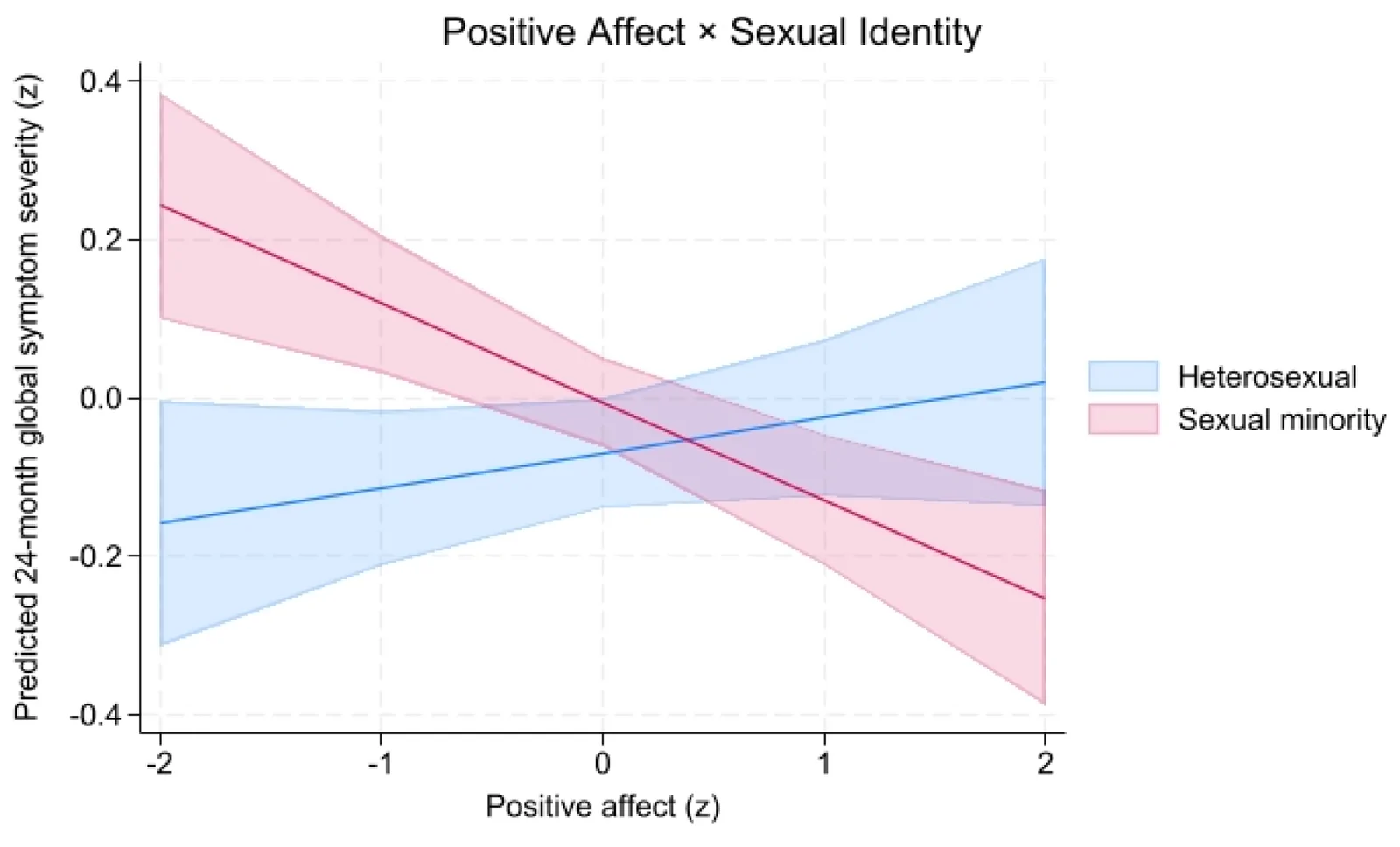
Interaction Between Recovering Positive Affect and Sexual Identity on Wave 5 Global Symptom Severity. Predicted standardized Wave 5 global symptom severity across levels of recovering positive affect by sexual identity from the fully adjusted moderation model. Shaded bands represent 95% confidence intervals.

In the sexual minority moderation model (Supplemental Table S14), Block 4 significantly improved model fit (ΔR^2^ = 0.014; F-change (12, 1543) = 3.01, *p* ≤ 0.001; final R^2^ = 0.409). Following adjustment for multiple testing, two interactions remained statistically significant: activism × sexual minority (adjusted *p* = 0.006) and recovering positive affect × sexual minority (adjusted *p* = 0.008) (Table 5). Simple slopes indicated that higher activism was associated with lower subsequent symptom severity among heterosexual youth but higher subsequent symptom severity among sexual minority youth (Table 6; Figure 1). Similarly, recovering positive affect demonstrated an inverse association with subsequent symptom severity among sexual minority youth that was not seen for heterosexual youth (Table 6; Figure 2).

## 4. Discussion

The purpose of the current study was to examine strengths across the RPM that may be associated with subsequent mental health symptoms. The findings of these exploratory secondary analyses supported the importance of strengths across the four domains of the model (regulatory, meaning-making, interpersonal and environmental). The study also investigated differences by sex at birth, gender, and sexual identity and found limited evidence that associations between strengths and subsequent mental health symptoms differed across the identity variables as operationalized in the current analyses. After adjustment for multiple testing, significant moderation was observed only for two strengths by sexual minority identity. Furthermore, across the full sample and subgroup analyses, structural and contextual stressors, including food insecurity, barriers to accessing mental health care, and cumulative adversity, were consistently associated with greater mental health symptoms when considered alone, although they became non-significant when strengths were added to the analyses. Further research examining the relationship between resilience and the material and structural conditions that shape young people’s lives is needed.

Patterns across identity groups further reinforce this point. Sexual and gender minoritized youth reported elevated symptoms, consistent with minority stress theory, which posits that stigma, discrimination, and chronic social stressors contribute to disparities in mental health (Baams et al., 2018; Green et al., 2022; Pellicane & Ciesla, 2022). In contrast, Black and Asian participants reported fewer symptoms, suggesting potential cultural, contextual, or measurement-related differences that warrant further investigation.

### 4.1. Regulatory and Meaning-Making Strengths

Findings from the overall resilience portfolio analyses suggest that not all strengths associated equally with mental health outcomes during adolescence and emerging adulthood, consistent with previous studies of the RPM. Regulatory strengths, including positive emotion regulation and self-compassion, were consistently related to lower symptoms across groups, again consistent with RPM research (Hamby et al., 2020b, 2025). Moderation analyses further suggested that recovering positive affect had a significant inverse association with subsequent symptoms among sexual minority youth while not producing a significant association for heterosexual participants. No robust moderation was observed for sex assigned at birth or gender identity. These findings reinforce the importance of internal regulatory capacities, not only for managing distress but also as an asset for sustaining positive emotional functioning under stress. Strategies like social–emotional learning programs (Durlak et al., 2022) taught in elementary and middle school may help enhance this group of strengths so that they are more available to potentially support mental health outcomes in later adolescence and young adulthood, and these findings support positive youth development models (Burkhard et al., 2020).

Interestingly, self-care activities, examined in an exploratory way, were associated with greater mental health symptoms perhaps because they are related to coping, and youth with greater concerns may be trying more self-care behaviors to manage their symptoms or may be encouraged to do so by professional helpers. In this sense, higher self-care counts may be better understood as indicators of help-seeking and coping activation in the presence of need. The finding may also reflect how self-care was operationalized. The measure captured the breadth of self-care strategies used rather than the frequency or effectiveness of individual strategies. Thus, a higher score may characterize youth trying a wider range of strategies in response to greater distress rather than indicating that self-care itself is associated with poorer outcomes. Future research should distinguish the breadth, frequency, and perceived effectiveness of self-care strategies. Together, these findings highlight the importance of distinguishing between the presence of coping efforts and their effectiveness and suggest that engaging in these behaviors does not necessarily indicate reduced risk, but rather may signal ongoing efforts to manage distress, differential exposure to adversity, or unmeasured contextual factors. The positive association observed here should therefore not be interpreted as evidence that self-care causes greater symptoms. These regulatory assets warrant further study.

Similarly, the role of the meaning-making strength of hope had a significant main effect overall and for individual symptom types and was not significant in moderator analyses. Having a sense of hope appears to be an important asset associated with lower mental health symptoms globally and across multiple symptom domains. This work supports recent studies of interventions to increase hope in young people through narrative exercises and meditation practices (Scioli et al., 2025). On the other hand, contrary to prior research (Hamby et al., 2020a; Hébert & Dassylva, 2026), purpose did not emerge as a significant correlate of lower mental health symptoms in the full sample. One explanation may be conceptual overlap with hope, which was highly correlated with the measure of purpose in the current study and demonstrated stronger associations with outcomes. However, multicollinearity diagnostics did not indicate problematic overlap in the fully adjusted model, suggesting that the null association for purpose was not simply attributable to excessive multicollinearity. This suggests that certain meaning-making strengths may operate in overlapping ways, with hope perhaps capturing more immediate, future-oriented processes relevant to mental health symptoms. More research is needed, as well as measurement innovations that more clearly distinguish hope from purpose.

It is notable that moderation analyses identified activism as one of only two strengths whose associations with later mental health differed by sexual identity. Whereas activism was associated with lower symptom severity among heterosexual youth, it was associated with higher symptom severity among sexual minority youth. Activism and purpose may go together and may be spurred by distress that comes from recognition of social problems and the need for social change. Youth who experienced minoritized identities may feel the need for activism more acutely. Research on one aspect of activism, critical consciousness, finds mixed results with mental health and well-being outcomes (Desmarais & Christophe, 2024; Maker Castro et al., 2022). Importantly, the current study did not distinguish between internal empowerment related to critical consciousness and activism involvement from the psychological toll or even burnout associated with navigating systemic oppression. These are important questions for future research. Engagement in activism often requires critical consciousness (Pinedo et al., 2024a, 2024b), which can increase exposure to and processing of structural inequities and may be related to distress. This pattern was specific to sexual minority youth and is consistent with models emphasizing the importance of minority strengths (Perrin et al., 2020) and minority stress contexts. The finding is consistent with previous work that finds variation in the impact of activism on well-being for different communities of young people and research describing how activism has both costs and benefits (Conner et al., 2023a). For example, a recent study found adolescents reported fewer costs to activism activities than emerging adults and gender- and sexual-identity-diverse young people reported greater costs but also more benefits (Conner et al., 2023b). The current study was limited in using an outcome focused on distress rather than well-being. Previous research on the RPM found unique associations of specific strengths with different outcomes. Indeed, at the bivariate level, activism was related to higher scores on many strengths including purpose and connectedness. Studies using dual-factor models show that well-being measures (differentiated from distress or symptom measures) are often higher among people who have robust strengths portfolios even when combined with high self-reported symptoms (Grych et al., 2020; Hamby et al., 2022). Activism behaviors may be such a dual-factor variable that contributes both to strengths but also to distress (Conner et al., 2023a, 2023b). Youth organizations seeking to promote youth voice and strengths through community organizing work should be aware of potential burnout but also of the ways that benefits co-exist with costs. Future research should help identify other aspects of young people’s resilience portfolios that might bolster well-being when involved in activism or measures of activism that separately measure the costs and benefits.

### 4.2. Interpersonal and Environmental Strengths

As has been found in other studies, interpersonal strengths showed mixed findings (Mitchell et al., 2021). Social support and group connectedness did not emerge as significantly associated with lower symptoms in multivariate models and, in some cases, were associated with higher symptoms. This finding is consistent with prior research showing mixed relationships between social support and well-being, particularly in adolescent populations (Mitchell et al., 2021). One explanation is that peer relationships during adolescence are complex and can serve as both sources of support and stress. Exposure to others’ distress, particularly within tightly connected peer networks, may contribute to processes such as social contagion (Martínez et al., 2023; Mitchell et al., 2025b; Wen et al., 2025) or compassion fatigue (Liao et al., 2022; Sakamoto, 2024). These dynamics may be especially pronounced among sexual and gender minority youth, who often have smaller, more interconnected social networks (Charmaraman et al., 2021) and have been found to be disproportionately exposed to peers experiencing similar stressors (Mitchell et al., 2021). In such contexts, efforts to seek support or engage in prevention conversations may coincide with higher levels of distress or may not provide sufficient buffering. These findings suggest that interpersonal strengths may operate in more context-dependent and indirect ways than commonly assumed and highlight the need for more nuanced approaches, including social network analyses, to understand when and for whom social connections are associated with better outcomes. Importantly, having a mentor was significantly related to lower symptoms both globally and across several symptom domains. This is consistent with research that mentors and mentoring programs like Big Brothers, Big Sisters can support resilience (Claro & Perelmiter, 2022; Herrera et al., 2023). Interpersonal strengths are clearly important but heterogeneous, suggesting the importance of measuring interpersonal strengths multidimensionally.

The current study also examined the environmental domain of the RPM (Banyard et al., 2025a), a relatively recent extension that emphasizes the role of place-based resources in resilience processes. The measure used in the current study was new and the lack of an association with global symptoms may suggest further refinement is needed, especially separating green and blue spaces from the built environment (Banyard et al., 2025b). At the domain level, greater community/environment resource use was associated with higher substance-use symptom counts after correction for multiple testing, whereas other domain-specific associations did not survive FDR correction. Given the unexpected direction of this association and the heterogeneous nature of the resources included in the measure, this finding warrants cautious interpretation and replication. This finding may reflect unmeasured characteristics such as the presence of unstructured socializing with risk-taking peers or third variables like urban density. Overall, the present findings provide limited evidence regarding the role of environmental resources in subsequent mental health symptoms. More research is needed.

Positive environmental assets, such as access to green spaces, safe community settings, and youth-oriented spaces like libraries (as opposed to risk factors like signs of physical decay), may promote resilience not merely by reducing risk, but by actively supporting restorative experiences, physical activity, and opportunities for positive social engagement and warrant more investigation (Jimenez et al., 2021; Kernaghan & Sturgeon, 2024) as part of ecological models of resilience (Ungar, 2011). We need to understand more about how access to spaces that enable restorative, social, and health-promoting activities support youth development. Despite growing interest in environmental influences (Li et al., 2025; Ma et al., 2023; Orsetti et al., 2022), such factors remain underrepresented in resilience research, suggesting that current models may underestimate the role of place-based resources in shaping youth well-being (Banyard et al., 2025b). Findings from the current study should be interpreted with caution given the need for measures that more clearly unpack “use” to understand whether it means access, engagement with these aspects of the environment, quality, and other dimensions. The current study represents a first and very preliminary step toward understanding the environmental domain.

### 4.3. Implications for Research, Practice, and Policy

Taken together, these findings extend the RPM in several important ways. First, they provide longitudinal evidence that strengths across multiple domains are prospectively associated with mental health symptoms above and beyond adversity and baseline mental health symptoms. Second, they suggest that the relative importance of domains is not uniform, with regulatory and meaning-making strengths showing significant associations with subsequent mental health symptoms during adolescence and emerging adulthood. Third, the mixed and context-dependent findings for interpersonal strengths indicate that resilience processes may be more conditional and dynamic than previously conceptualized. Finally, although there was limited evidence of moderation overall, the two significant interactions involving sexual minority identity highlight the importance of considering resilience within the context of minority stress and broader social and structural inequities.

These findings have several implications for research, practice, and policy. First, clinicians and youth-serving professionals should incorporate systematic assessment of strengths across multiple domains, particularly regulatory capacities and meaning-making assets, when working with adolescents and emerging adults. A strengths-based approach that goes beyond symptom reduction to include assets such as emotion regulation, hope, and access to supportive environments, as well as resources like mentors, may better capture pathways to resilience. The current study extends work on Minority Strengths Theory, developed using adult samples, that showed how social connections, identity pride, and self esteem can be associated with mental health (Perrin et al., 2020).

The heterogeneous and context-dependent findings for interpersonal strengths highlight the importance of tailoring interventions to developmental stage and social context. Programs that rely heavily on peer support or support-seeking should consider the quality, structure, and potential burdens of social networks, particularly for sexual and gender minority youth, whose support systems may be more constrained or carry additional emotional demands. Connecting work on resilience with research on informal helping and youth development will be important for future research (Lynch et al., 2023).

The observed differences in associations across identity groups were limited, but the findings reinforce the importance of culturally responsive and identity-affirming approaches to prevention and intervention. Strategies that are effective for one group of youth may not generalize to others, particularly in the context of minority stress and structural inequities. Interventions should be designed with attention to the unique strengths, stressors, and social contexts of diverse youth populations.

Finally, these findings have implications for resilience theory and measurement. The results are consistent with multidimensional models such as the Resilience Portfolio Model (Grych et al., 2015), while also suggesting that the relative importance of different domains may vary across developmental stages and contexts. Future research should continue to examine how strengths interact across domains, incorporate underexplored constructs such as environmental assets, and include both distress-based and positive well-being outcomes to more fully capture resilience processes.

### 4.4. Strengths, Limitations, and Future Directions

This study has limitations that should be considered. Because the study included numerous moderation analyses, interactions were evaluated using the Benjamini–Hochberg false discovery rate procedure to reduce the likelihood of Type I error. Following correction, only two interactions remained statistically significant, suggesting that subgroup differences should be interpreted cautiously until replicated. The domain-specific analyses similarly involved multiple comparisons; FDR correction was therefore applied across the 120 strength-by-domain tests, and only corrected associations were interpreted as statistically significant. Mental health symptoms were the sole outcome; future research should include positive indicators of well-being to capture a fuller picture of resilience. Some constructs (e.g., mentoring) were measured with brief indicators that may not fully capture their complexity. The self-care measure was operationalized as a count of self-care behaviors rather than a traditional psychometric scale. Accordingly, internal consistency is of more limited interpretive value because different self-care behaviors are not necessarily expected to co-occur. Nevertheless, the measure demonstrated only modest internal consistency, and findings involving self-care should be interpreted cautiously.

Strengths were assessed at different time points due to constraints of the secondary study design, which may influence observed associations and limit conclusions about temporal ordering. Because each strength was assessed at a specific wave rather than repeatedly across waves, strength construct and measurement timing could not be disentangled; differences in associations across strengths may therefore partly reflect their differing proximity to the Wave 5 outcome. Future research that measures the same strengths at multiple time points is needed to distinguish construct-specific associations from time-varying effects.

All measures were based on participant self-report and may be affected by shared method variance, recall, or reporting biases. Although models adjusted for baseline mental health symptoms, adversity, demographic characteristics, and multiple social determinants of health, residual confounding by unmeasured individual, family, peer, or contextual factors remains possible. Accordingly, the prospective associations observed here should not be interpreted as causal effects of strengths on subsequent mental health. For example, youth experiencing greater distress may be more likely to report both challenges and coping behaviors (e.g., support-seeking, self-care), which may partially explain findings in which some strengths were associated with higher symptom levels.

The complete-case analytic sample represented 47.1% of the qualifying longitudinal cohort, raising the possibility of selection bias due to attrition and missing data across the staggered assessments. If youth experiencing greater distress or possessing fewer strengths were less likely to remain in the study or to have complete data, such differential inclusion could bias the magnitude of the observed associations and limit their generalizability. However, findings from the inverse-probability-of-analytic-sample-inclusion weighted sensitivity analysis were substantively similar to the primary complete-case findings, providing some evidence that the primary results were robust to selection associated with the measured baseline characteristics used to construct the weights. The IPW sensitivity analysis addresses differential inclusion associated with the measured baseline characteristics included in the weighting model but cannot account for selection associated with unmeasured factors or potentially relevant time-varying characteristics occurring during follow-up. Furthermore, participants were recruited through social media and were required to complete surveys in English, which may limit generalizability to youth with less access to technology or from non-English-speaking backgrounds. The large sample of gender- and sexual-identity-diverse young people is a strength but the exploratory nature of the current analyses and the smaller sample sizes for different identities of young people within these broad binary variables is a limitation. Future work is needed to better examine variation among sexual and gender minority groups.

Finally, some measures, particularly within the environmental domain, were newly developed for this study. Although these measures were grounded in prior research and demonstrated acceptable reliability, additional validation is needed. Future research should continue to refine measurement of environmental strengths and examine how resilience processes unfold across contexts using designs that can better capture dynamic and potentially bidirectional relationships. Separating built environment from green and blue spaces will also be important in future measurement.

## 5. Conclusions

This study contributed to a growing body of resilience research by demonstrating that strengths across multiple domains were associated with subsequent mental health symptoms among adolescents and emerging adults, while also highlighting some variation in these associations across contexts and limited variation across identity groups. Findings were consistent with the view that resilience is not a fixed trait or a uniform process, but a dynamic and multidimensional system shaped by developmental stage, social position, and access to resources. In particular, the significant effects for regulatory and meaning-making strengths suggested that a variety of internal capacities may be particularly relevant to youth mental health. At the same time, mixed and context-dependent findings for interpersonal and environmental strengths highlight the need for more nuanced, contextually grounded models of resilience and more research on external resources and their associations with well-being. Overall, these results highlight the value of more comprehensive, equity-informed approaches to resilience that integrate individual, relational, and structural factors. Future research should examine how youths’ internal capacities, access to supportive environments, and broader social conditions jointly shape opportunities for resilience and well-being.

## Figures and Tables

**Table 1:** Characteristics of the qualifying longitudinal cohort and comparison of participants included versus excluded from the final complete-case analytic sample.

Characteristic	Qualifying Cohort (N = 3360)	Final Analytic Sample (N = 1584)	Excluded from Analytic Sample (N = 1776)	*p* Value
Demographics				
Adolescent	1867 (55.6)	877 (55.4)	990 (55.7)	0.83
Female	2068 (61.5)	1057 (66.7)	1011 (56.9)	<0.001
Any sexual minority	1947 (57.9)	892 (56.3)	1055 (59.4)	0.07
Any gender minority	1206 (35.9)	535 (33.8)	671 (37.8)	0.016
White	2474 (73.6)	1138 (71.8)	1336 (75.2)	0.005
Black	399 (11.9)	202 (12.8)	197 (11.1)	0.16
Asian	420 (12.5)	220 (13.9)	200 (11.3)	0.020
American Indian/Alaska Native	124 (3.7)	62 (3.9)	62 (3.5)	0.54
Hispanic/Latino	719 (21.4)	328 (20.7)	391 (22.0)	0.36
Baseline characteristics, Mean (SD)				
Age (years)	17.52 (2.23)	17.55 (2.23)	17.49 (2.24)	0.41
Global symptom severity (z)	0.00 (1.00)	−0.04 (0.99)	0.03 (1.01)	0.001
Adversity (z)	−0.05 (0.98)	−0.08 (0.96)	−0.02 (1.00)	0.07
Food insecurity (z)	0.00 (1.00)	0.00 (1.02)	0.00 (0.98)	0.81
Financial instability (z)	0.00 (1.00)	−0.05 (0.97)	0.04 (1.02)	0.004
Mental health care barriers (z)	0.00 (1.00)	0.01 (1.00)	−0.01 (1.00)	0.57
Poor home conditions (z)	−0.02 (0.96)	−0.03 (0.98)	−0.01 (0.95)	0.55
Neighborhood disorder (z)	0.00 (1.00)	−0.01 (1.01)	0.01 (0.99)	0.62
Discrimination (z)	0.00 (1.00)	−0.03 (0.99)	0.03 (1.01)	0.10

Note. Values are N (%) unless otherwise indicated. Continuous variables are presented as mean (SD). Adolescents were aged 13–17 years and young adults were aged 18–22 years at baseline. The excluded group comprises participants from the qualifying longitudinal cohort who were omitted from the primary analyses because of missing data required for complete-case estimation. *p* values compare participants included in the final analytic sample with those excluded from the final analytic sample. Baseline symptom severity and social determinant variables are standardized (*z*) scores. Hispanic/Latino ethnicity was assessed separately from race; therefore, Hispanic/Latino ethnicity and the racial categories shown are not mutually exclusive.

**Table 2:** Correlations among strengths, adversity and mental health symptoms.

	1	2	3	4	5	6	7	8	9	10	11	12	13	14	15
1. Global mental health (Wave 5)	1.0	---	---	---	---	---	---	---	---	---	---	---	---	---	---
2. Suicide Prevention	0.02	1.0	---	---	---	---	---	---	---	---	---	---	---	---	---
3. Mentor	−0.09 ***	0.04	1.0	---	---	---	---	---	---	---	---	---	---	---	---
4. Activism	0.12 ***	0.10 ***	0.13 ***	1.0	---	---	---	---	---	---	---	---	---	---	---
5. Self-care	0.17 ***	0.15 ***	0.19 ***	0.31 ***	1.0	---	---	---	---	---	---	---	---	---	---
6. Social support receipt	−0.03	0.06 **	0.22 ***	0.13 ***	0.16 ***	1.0	---	---	---	---	---	---	---	---	---
7. Social support seeking	−0.02	0.09 ***	0.15 ***	0.12 ***	0.14 ***	0.49 ***	1.0	---	---	---	---	---	---	---	---
8. Hope	−0.38 ***	0.01	0.20 ***	0.04	0.08 ***	0.24 ***	0.23 ***	1.0	---	---	---	---	---	---	---
9. Purpose	−0.30 ***	0.02	0.26 ***	0.09 ***	0.13 ***	0.28 ***	0.26 ***	0.74 ***	1.0	---	---	---	---	---	---
10. Connectedness	−0.07 ***	0.05 *	0.17 ***	0.07 ***	0.12 ***	0.24 ***	0.25 ***	0.20 ***	0.26 ***	1.0	---	---	---	---	---
11. Self-compassion	−0.31 ***	−0.01	0.11 ***	0.07 **	0.11 ***	0.25 ***	0.28 ***	0.50 ***	0.45 ***	0.21 ***	1.0	---	---	---	---
12. Built environment	−0.08 ***	0.09 ***	0.10 ***	0.15 ***	0.19 ***	0.13 ***	0.12 ***	0.20 ***	0.24 ***	0.14 ***	0.16 ***	1.0	---	---	---
13. Positive emotional reg	−0.28 ***	0.002	0.16 ***	0.03	0.09 ***	0.15 ***	0.13 ***	0.54 ***	0.47 ***	0.16 ***	0.47 ***	0.17 ***	1.0	---	---
14. Global mental health (Wave 1)	0.58 ***	0.03	−0.05 **	0.15 ***	0.17 ***	−0.02	−0.01	−0.38 ***	−0.31 ***	−0.10 ***	−0.31 ***	−0.11 ***	−0.29 ***	1.0	---
15. Adversity (Wave 1)	0.30 ***	0.03	−0.05 *	0.13 ***	0.17 ***	−0.04	−0.01	−0.17 ***	−0.14 ***	−0.08 ***	−0.12 ***	−0.07 ***	−0.10 ***	0.33 ***	1.0

Note. *** *p* ≤ 0.001, ** *p* ≤ 0.01, * *p* ≤ 0.05.

**Table 3:** Hierarchical regression examining Wave 5 global symptom severity (N = 1584).

Construct	Model 1: Demographics and SDoH		Model 2: +Baseline Mental Health and Adversity		Model 3: +Strengths	
	B (SE)	*p* Value	B (SE)	*p* Value	B (SE)	*p* Value
Demographics/Identity/SDOH						
Female at birth	0.116 (0.048)	0.015	0.019 (0.043)	0.664	−0.029 (0.043)	0.503
Age	0.000 (0.010)	0.969	0.006 (0.009)	0.538	0.007 (0.009)	0.454
Sexual minority identity	0.169 (0.056)	0.002	0.085 (0.050)	0.088	0.043 (0.049)	0.379
Gender minority identity	0.259 (0.060)	<0.001	0.140 (0.054)	0.009	0.085 (0.052)	0.105
Black race	0.050 (0.068)	0.459	0.065 (0.061)	0.281	0.105 (0.059)	0.074
Asian race	−0.091 (0.066)	0.169	−0.010 (0.059)	0.870	0.008 (0.057)	0.891
American Indian race	−0.051 (0.116)	0.659	−0.183 (0.104)	0.079	−0.191 (0.101)	0.059
Latino ethnicity	0.056 (0.056)	0.318	0.083 (0.050)	0.098	0.069 (0.049)	0.155
Food insecurity	0.084 (0.026)	0.001	0.032 (0.023)	0.172	0.031 (0.023)	0.167
Financial instability	0.032 (0.027)	0.248	−0.011 (0.025)	0.670	−0.013 (0.024)	0.601
Mental health care barriers	0.115 (0.024)	<0.001	0.025 (0.022)	0.243	0.005 (0.021)	0.803
Poor home conditions	0.051 (0.025)	0.044	0.033 (0.023)	0.148	0.033 (0.022)	0.140
Neighborhood disorder	0.018 (0.024)	0.451	−0.030 (0.022)	0.170	−0.025 (0.021)	0.238
Discrimination exposure	0.164 (0.024)	<0.001	0.036 (0.022)	0.107	0.034 (0.022)	0.113
Baseline Mental Health/Adversity						
Baseline global symptoms	—	—	0.463 (0.024)	<0.001	0.375 (0.025)	<0.001
Baseline adversity	—	—	0.078 (0.025)	0.002	0.068 (0.024)	0.005
Strengths						
Suicide prevention receipt	—	—	—	—	0.067 (0.065)	0.309
Had a mentor	—	—	—	—	−0.120 (0.041)	0.004
Activism	—	—	—	—	0.016 (0.021)	0.441
Self-care	—	—	—	—	0.087 (0.022)	<0.001
Received social support	—	—	—	—	−0.001 (0.029)	0.978
Sought social support	—	—	—	—	−0.010 (0.035)	0.776
Hope	—	—	—	—	−0.159 (0.031)	<0.001
Purpose	—	—	—	—	0.027 (0.030)	0.368
Group connection	—	—	—	—	0.039 (0.020)	0.052
Self-compassion	—	—	—	—	−0.098 (0.024)	<0.001
Use of community/environment resources	—	—	—	—	0.005 (0.020)	0.791
Recovering positive affect	---	---	---	---	-0.048 (0.024)	0.047
Model Fit						
R^2^	0.175		0.345		0.395	
ΔR^2^	—		0.170		0.050	
F (df)	23.73 (14, 1569)	<0.001	203.50 (2, 1567)	<0.001	10.77 (12, 1555)	<0.001

**Table 4:** Block 3 strengths across Wave 5 mental health outcomes.

Strength	Somatic	Sleep	Inattention	Depression	Anger	Mania	Anxiety	Psychosis	Repetitive	Substance Count
Suicide prevention receipt	0.003	0.068	0.164	0.100	0.107	−0.099	0.080	−0.082	0.032	0.053
Had a mentor	**−0.149**	−0.060	**−0.152**	−0.094	−0.091	0.026	**−0.** **116**	−0.013	−0.066	−0.030
Activism	0.029	−0.012	0.029	−0.015	0.008	−0.017	0.043	0.030	0.004	0.007
Self-care	**0.095**	**0.093**	**0.078**	**0.074**	**0.070**	0.066	**0.070**	0.018	0.057	−0.016
Received social support	0.043	0.018	−0.033	−0.026	0.025	−0.034	0.024	−0.012	0.001	−0.016
Sought social support	−0.004	0.010	0.031	0.007	−0.071	0.001	−0.036	−0.001	−0.000	0.016
Hope	**−0.115**	**−0.125**	−0.087	**−0.159**	−0.068	−0.072	**−0.133**	**−0.177**	**−0.107**	−0.027
Purpose	0.043	0.055	0.001	0.015	0.027	−0.027	0.064	0.013	0.035	−0.077
Group connectedness	0.033	0.027	0.008	0.029	0.025	0.006	0.045	0.044	0.011	0.024
Self-compassion	**−0.074**	−0.040	**−0.103**	**−0.124**	**−0.152**	−0.050	**−0.161**	−0.011	−0.005	0.050
Use Community/environment	−0.013	−0.039	0.024	−0.046	−0.053	0.023	−0.021	0.012	0.032	**0.104**
Recovering positive affect	−0.003	−0.069	−0.043	**−0.071**	**−0.085**	0.027	**−0.090**	0.022	−0.062	0.052

Note. Values are unstandardized regression coefficients (B) from the final nested model (Block 3), adjusting for demographics, social determinants of health, discrimination exposure, baseline symptom severity, and adversity. Outcomes were standardized (z-scored) prior to analysis; thus, coefficients represent change in standard deviation units of the outcome. N = 1584 for all models. Bold indicates associations that remained statistically significant following Benjamini–Hochberg false discovery rate correction across the 120 strength-by-domain tests (12 strengths × 10 symptom domains).

**Table 5:** Strength × identity interactions with Wave 5 global mental health symptoms.

Strength	Sex Assigned at Birth B (SE)	FDR *p*	Gender Minority B (SE)	FDR *p*	Sexual Minority B (SE)	FDR *p*
Suicide prevention receipt	0.113 (0.136)	0.614	−0.099 (0.157)	0.720	−0.067 (0.130)	0.752
Had a mentor	0.002 (0.087)	0.983	0.110 (0.086)	0.466	0.039 (0.083)	0.752
Activism	0.017 (0.041)	0.757	0.100 (0.043)	0.142	**0.157 (0.041)**	0.006
Self-care	0.084 (0.044)	0.231	−0.071 (0.047)	0.425	−0.019 (0.042)	0.752
Received social support	0.071 (0.059)	0.480	−0.057 (0.058)	0.514	−0.067 (0.059)	0.502
Sought social support	−0.100 (0.093)	0.502	0.192 (0.121)	0.407	0.199 (0.102)	0.231
Hope	−0.004 (0.067)	0.983	−0.040 (0.065)	0.720	−0.022 (0.063)	0.794
Purpose	−0.081 (0.064)	0.466	0.161 (0.064)	0.104	0.078 (0.061)	0.466
Social connectedness	0.020 (0.043)	0.752	0.033 (0.041)	0.621	−0.002 (0.040)	0.983
Self-compassion	−0.053 (0.050)	0.502	0.073 (0.053)	0.466	0.047 (0.048)	0.514
Use Community/environment	−0.055 (0.042)	0.466	−0.099 (0.045)	0.169	−0.043 (0.040)	0.502
Recovering positive affect	0.102 (0.052)	0.231	−0.136 (0.053)	0.104	**−0.168 (0.048)**	0.008
	ΔR^2^	Block *p*	ΔR^2^	Block *p*	ΔR^2^	Block *p*
Interaction block	0.006	0.256	0.013	0.001	0.014	<0.001

Note. Values are interaction coefficients (B) with standard errors in parentheses. Interaction coefficients indicate how much the association between a given strength and Wave 5 global mental health symptoms differs for the indicated identity group relative to its reference group; they are not the association between the strength and symptoms within that identity group. *p* values shown for individual interactions are Benjamini–Hochberg FDR-adjusted across the 36 interaction tests. Bold indicates interactions that remained statistically significant after FDR correction. ΔR^2^ and Block *p* values refer to the joint test of the 12 interaction terms for each moderator. Full nested regression models are presented in Supplemental Tables S12–S14.

**Table 6:** Simple slopes for significant strength × sexual minority identity interactions with Wave 5 global mental health symptoms.

Strength	Sexual Identity	B	SE	95% CI	*p*
Activism	Heterosexual	−0.071	0.031	−0.132, −0.010	0.023
Activism	Sexual minority	0.086	0.028	0.031, 0.141	0.002
Recovering positive affect	Heterosexual	0.045	0.036	−0.025, 0.114	0.208
Recovering positive affect	Sexual minority	−0.124	0.032	−0.187, −0.060	<0.001

Note. Values are estimated marginal effects (simple slopes; dy/dx) from the fully adjusted sexual minority identity moderation model. B represents the estimated association between each standardized strength and standardized Wave 5 global mental health symptom severity within each sexual identity group. Models adjusted for demographics, social determinants of health, discrimination exposure, baseline global mental health symptoms, adversity, and all strengths and strength × sexual minority identity interaction terms. N = 1584. CI = confidence interval.

## Data Availability

Data will be available through the NIH Data Archive upon study completion (July 2027). Earlier access is possible upon reasonable request to the corresponding author.

## Supplementary Materials

The following supporting information can be downloaded at https://www.mdpi.com/article/10.3390/bs16101896/s1, Table S1: Inverse-Probability-Weighted Fully Adjusted Model of Wave 5 Global Mental Health Symptoms; Table S2: Nested Linear Regression Examining Wave 5 Somatic Symptoms (N = 1584); Table S3: Nested Linear Regression Examining Wave 5 Sleep Symptoms (N = 1584); Table S4: Nested Linear Regression Examining Wave 5 Inattention Symptoms (N = 1584); Table S5: Nested Linear Regression Examining Wave 5 Depression Symptoms (N = 1584); Table S6: Nested Linear Regression Examining Wave 5 Anger/Irritability Symptoms (N = 1584); Table S7: Nested Linear Regression Examining Wave 5 Mania/Hypomania Symptoms (N = 1584); Table S8: Nested Linear Regression Examining Wave 5 Anxiety Symptoms (N = 1584); Table S9: Nested Linear Regression Examining Wave 5 Psychosis Symptoms (N = 1584); Table S10: Nested Linear Regression Examining Wave 5 Repetitive Thoughts/Behaviors (N = 1584); Table S11: Nested Linear Regression Examining Wave 5 Substance Use Count (N = 1584); Table S12: Nested Regression With Sex Assigned at Birth Moderation of Strengths on Global Mental Health at Wave 5; Table S13: Nested Regression Examining Gender Identity Moderation of Strengths on Wave 5 Global Mental Health Symptoms (N = 1584); Table S14: Nested Regression With Sexual Identity Moderation of Strength Associations With Global Mental Health at Wave 5.
