# Supplementary material for: Understanding How Multiple Strengths Support the Mental Health of Adolescents and Young Adults in a Gender-Diverse Sample
Source: Behav Sci (Basel). 2026 Oct 14;16(10):1896. doi: 10.3390/bs16101896 (PMC13701975; doi:10.3390/bs16101896)
Supplement: Supplementary file 1 [file behavsci-16-01896-s001.zip › behavsci-4515535-supplementary.pdf]

Supplemental Table S1

Inverse-Probability-Weighted Fully Adjusted Model of Wave 5 Global Mental Health Symptoms

Variable	B	SE	95% CI	p
Sex assigned at birth: female	-0.021	.044	-0.107, 0.065	.633
Age	0.008	.009	-0.009, 0.025	.367
Sexual minority identity	0.046	.049	-0.050, 0.143	.344
Gender minority identity	0.086	.053	-0.017, 0.189	.103
Black	0.110	.068	-0.024, 0.245	.107
Asian	0.013	.057	-0.099, 0.125	.821
American Indian/Alaska Native	-0.187	.109	-0.401, 0.027	.086
Hispanic/Latino	0.073	.050	-0.026, 0.172	.147
Food insecurity	0.034	.024	-0.014, 0.082	.160
Financial instability	-0.014	.023	-0.060, 0.031	.537
Mental health care barriers	0.002	.022	-0.041, 0.044	.942
Residential instability	0.033	.023	-0.012, 0.079	.149
Poor home conditions	-0.024	.021	-0.066, 0.017	.251
Discrimination	0.036	.023	-0.009, 0.081	.117
Baseline global mental health symptoms	0.374	.029	0.317, 0.431	<.001
Baseline adversity	0.066	.027	0.014, 0.119	.013
Suicide prevention receipt	0.066	.077	-0.085, 0.218	.390
Mentor	-0.118	.042	-0.200, -0.036	.005
Activism	0.018	.024	-0.028, 0.064	.447
Self-care	0.081	.024	0.034, 0.129	.001
Social support receipt	-0.005	.028	-0.060, 0.050	.863
Social support seeking	-0.005	.032	-0.068, 0.059	.883
Hope	-0.163	.037	-0.235, -0.091	<.001
Purpose	0.035	.032	-0.027, 0.097	.270
Connectedness	0.043	.021	0.002, 0.083	.038
Self-compassion	-0.094	.027	-0.147, -0.041	.001
Community/environmental resource use	0.007	.022	-0.037, 0.051	.750
Recovering positive affect	-0.059	.027	-0.111, -0.007	.027

Note. N = 1,584. B values are unstandardized regression coefficients. The fully adjusted model was estimated using stabilized inverse-probability-of-analytic-sample-inclusion weights; probability weights yield robust standard errors. The weighting model was estimated among the qualifying longitudinal cohort (N = 3,360) using baseline age, sex assigned at birth, sexual minority identity, gender minority identity, race/ethnicity, food insecurity, financial instability, mental health care barriers, residential instability, poor home conditions, discrimination, global mental health symptoms, and adversity. Stabilized weights had mean = 1.00, 99th percentile = 1.47, and maximum = 1.96.

Supplemental Table S2

Nested Linear Regression Examining Wave 5 Somatic Symptoms (N = 1,584)

Construct	B	SE	β	P value	FDR-adjusted P value
**Demographics / Identity / SDOH**					
Female	0.118	0.047	0.118	0.013	—
Age	0.010	0.010	0.010	0.288	—
Sexual minority	0.047	0.053	0.047	0.380	—
Gender minority	0.074	0.057	0.074	0.193	—
Black	0.083	0.064	0.083	0.193	—
Asian	−0.020	0.063	−0.020	0.750	—
American Indian	−0.136	0.110	−0.136	0.217	—
Latino	0.007	0.053	0.007	0.899	—
Food insecurity	0.049	0.025	0.049	0.045	—
Financial instability	−0.021	0.026	−0.021	0.423	—
Mental health care barriers	0.025	0.023	0.025	0.266	—
Poor home conditions	0.021	0.024	0.021	0.387	—
Neighborhood disorder	−0.003	0.023	−0.003	0.901	—
Discrimination	0.012	0.023	0.012	0.615	—
**Baseline mental health / adversity**					
Somatic	0.378	0.024	0.378	< .001	—
Adversity	0.055	0.026	0.055	0.037	—
**Strengths**					
Suicide prevention exposure	0.003	0.071	0.003	0.970	.993
**Mentor**	**−0.149**	**0.045**	**−0.149**	**0.001**	**.008**
Activism	0.029	0.023	0.029	0.205	.425
**Self-care**	**0.095**	**0.024**	**0.095**	**< .001**	**.001**
Support receipt	0.043	0.031	0.043	0.167	.369
Support seeking	−0.004	0.038	−0.004	0.906	.954
**Hope**	**−0.115**	**0.034**	**−0.115**	**0.001**	**.007**
Purpose in life	0.043	0.033	0.043	0.193	.414
Social connectedness	0.033	0.022	0.033	0.130	.313
**Self-compassion**	**−0.074**	**0.026**	**−0.074**	**0.005**	**.027**
Use of healthy built environment	−0.013	0.022	−0.013	0.546	.721
Positive affect	−0.003	0.026	−0.003	0.905	.954

Note. Unstandardized regression coefficients (B) and standardized coefficients (β) from the final 3-block model. Outcome standardized (z). Benjamini–Hochberg false discovery rate (FDR) correction was applied across the 120 strength-by-domain tests (12 strengths × 10 symptom domains); FDR-adjusted p values are therefore reported only for the strength coefficients. Bold indicates strength associations that remained statistically significant after FDR correction (pFDR < .05).

Model fit: Block 1: $R^2 = 0.144$, Block 2: $R^2 = 0.289$, Block 3: $R^2 = 0.316$; ΔR^2 (Block 3) = 0.027

Supplemental Table S3

Nested Linear Regression Examining Wave 5 Sleep Symptoms (N = 1,584)

Construct	B	SE	β	P value	FDR-adjusted P value
**Demographics / Identity / SDOH**					
Female	−0.020	0.049	−0.020	0.679	—
Age	−0.009	0.010	−0.009	0.393	—
Sexual minority	0.062	0.056	0.062	0.268	—
Gender minority	0.058	0.060	0.058	0.336	—
Black	0.022	0.068	0.022	0.746	—
Asian	−0.027	0.066	−0.027	0.680	—
American Indian	0.095	0.117	0.095	0.417	—
Latino	−0.003	0.056	−0.003	0.960	—
Food insecurity	0.052	0.026	0.052	0.046	—
Financial instability	−0.036	0.028	−0.036	0.203	—
Mental health care barriers	0.023	0.024	0.023	0.340	—
Poor home conditions	0.043	0.025	0.043	0.092	—
Neighborhood disorder	−0.013	0.024	−0.013	0.587	—
Discrimination	0.024	0.025	0.024	0.328	—
**Baseline mental health / adversity**					
Sleep	0.317	0.025	0.317	< .001	—
Adversity	0.077	0.028	0.077	0.006	—
**Strengths**					
Suicide prevention exposure	0.068	0.075	0.020	0.366	.578
Mentor	−0.060	0.048	−0.029	0.211	.427
Activism	−0.012	0.024	−0.012	0.627	.775
**Self-care**	**0.093**	**0.025**	**0.093**	**< .001**	**.003**
Support receipt	0.018	0.033	0.018	0.584	.762
Support seeking	0.010	0.040	0.010	0.806	.896
**Hope**	**−0.125**	**0.036**	**−0.125**	**0.001**	**.005**
Purpose in life	0.055	0.035	0.055	0.114	.292
Social connectedness	0.027	0.023	0.027	0.248	.471
Self-compassion	−0.040	0.028	−0.040	0.147	.339
Use of healthy built environment	−0.039	0.023	−0.039	0.100	.261
Positive affect	−0.069	0.028	−0.069	0.013	.058

Note. Unstandardized regression coefficients (B) and standardized coefficients (β) from the final 3-block model. Outcome standardized (z). Benjamini–Hochberg false discovery rate (FDR) correction was applied across the 120 strength-by-domain tests (12 strengths × 10 symptom domains); FDR-adjusted p values are reported only for the strength coefficients. Bold indicates strength associations that remained statistically significant after FDR correction ($p_{FDR} < .05$).

Model fit: Block 1: $R^2 = 0.100$, Block 2: $R^2 = 0.215$, Block 3: $R^2 = 0.244$; ΔR^2 (Block 3) = 0.030

Supplemental Table S4

Nested Linear Regression Examining Wave 5 Inattention Symptoms (N = 1,584)

Construct	B	SE	β	P value	FDR-adjusted P value
**Demographics / Identity / SDOH**					
Female	−0.045	0.050	−0.045	0.366	—
Age	0.000	0.010	0.000	0.970	—
Sexual minority	0.031	0.058	0.031	0.588	—
Gender minority	0.048	0.062	0.048	0.433	—
Black	0.051	0.069	0.051	0.461	—
Asian	0.035	0.067	0.035	0.608	—
American Indian	−0.158	0.119	−0.158	0.184	—
Latino	0.029	0.057	0.029	0.615	—
Food insecurity	0.007	0.026	0.007	0.793	—
Financial instability	−0.006	0.029	−0.006	0.841	—
Mental health care barriers	0.056	0.024	0.056	0.023	—
Poor home conditions	0.038	0.026	0.038	0.142	—
Neighborhood disorder	−0.022	0.025	−0.022	0.368	—
Discrimination	−0.011	0.025	−0.011	0.658	—
**Baseline mental health / adversity**					
Inattention	0.297	0.025	0.297	< .001	—
Adversity	0.011	0.028	0.011	0.695	—
**Strengths**					
Suicide prevention exposure	0.164	0.077	0.164	0.034	.136
**Mentor**	**−0.152**	**0.049**	**−0.152**	**0.002**	**.012**
Activism	0.029	0.025	0.029	0.247	.463
**Self-care**	**0.078**	**0.026**	**0.078**	**0.002**	**.012**
Support receipt	0.018	0.033	0.018	0.584	.748
Support seeking	0.010	0.040	0.010	0.806	.895
Hope	−0.087	0.037	−0.087	0.018	.074
Social connectedness	0.027	0.024	0.027	0.260	.475
**Self-compassion**	**−0.103**	**0.028**	**−0.103**	**< .001**	**.003**
Use of healthy built environment	−0.039	0.023	−0.039	0.098	.261
Positive affect	−0.069	0.028	−0.069	0.013	.058

Note. Unstandardized regression coefficients (B) and standardized coefficients (β) from the final 3-block model. Outcome standardized (z). Benjamini–Hochberg false discovery rate (FDR) correction was applied across the 120 strength-by-domain tests (12 strengths × 10 symptom domains); FDR-adjusted p values are therefore reported only for the strength coefficients. Bold indicates strength associations that remained statistically significant after FDR correction (pFDR < .05).

Model fit: Block 1: $R^2 = 0.076$, Block 2: $R^2 = 0.175$, Block 3: $R^2 = 0.215$; ΔR^2 (Block 3) = 0.041.

Supplemental Table S5

Nested Linear Regression Examining Wave 5 Depression Symptoms (N = 1,584)

Construct	B	SE	β	P value	FDR-adjusted P value
**Demographics / Identity / SDOH**					
Female	−0.041	0.047	−0.041	0.390	—
Age	0.010	0.010	0.010	0.317	—
Sexual minority	0.037	0.054	0.037	0.500	—
Gender minority	0.091	0.058	0.091	0.118	—
Black	0.080	0.065	0.080	0.220	—
Asian	−0.092	0.064	−0.092	0.149	—
American Indian	−0.279	0.113	−0.279	0.013	—
Latino	0.015	0.054	0.015	0.789	—
Food insecurity	0.065	0.025	0.065	0.010	—
Financial instability	−0.041	0.027	−0.041	0.130	—
Mental health care barriers	0.019	0.023	0.019	0.428	—
Poor home conditions	0.038	0.024	0.038	0.122	—
Neighborhood disorder	−0.032	0.023	−0.032	0.173	—
Discrimination	0.036	0.024	0.036	0.132	—
**Baseline mental health / adversity**					
Depression	0.275	0.025	0.275	< .001	—
Adversity	0.047	0.027	0.047	0.080	—
**Strengths**					
Suicide prevention exposure	0.100	0.073	0.100	0.169	.369
Mentor	−0.094	0.046	−0.094	0.040	.139
Activism	−0.015	0.023	−0.015	0.513	.687
**Self-care**	**0.074**	**0.024**	**0.074**	**0.003**	**.016**
Support receipt	−0.026	0.032	−0.026	0.419	.640
Support seeking	0.007	0.039	0.007	0.856	.926
**Hope**	**−0.159**	**0.035**	**−0.159**	**< .001**	**.0001**
Purpose in life	0.015	0.034	0.015	0.649	.787
Social connectedness	0.029	0.022	0.029	0.197	.415
**Self-compassion**	**−0.124**	**0.027**	**−0.124**	**< .001**	**.0001**
Use of healthy built environment	−0.046	0.023	−0.046	0.040	.139
**Positive affect**	**−0.071**	**0.027**	**−0.071**	**0.009**	**.045**

Note. Unstandardized regression coefficients (B) and standardized coefficients (β) from the final 3-block model. Outcome standardized (z). Benjamini–Hochberg false discovery rate (FDR) correction was applied across the 120 strength-by-domain tests (12 strengths × 10 symptom domains); FDR-adjusted p values are therefore reported only for the strength coefficients. Bold indicates strength associations that remained statistically significant after FDR correction (pFDR < .05).

Model fit: Block 1: $R^2 = 0.110$, Block 2: $R^2 = 0.233$, Block 3: $R^2 = 0.300$; ΔR^2 (Block 3) = 0.068.

Supplemental Table S6

Nested Linear Regression Examining Wave 5 Anger/Irritability Symptoms (N = 1,584)

Construct	B	SE	β	P value	FDR-adjusted P value
**Demographics / Identity / SDOH**					
Female	0.047	0.049	0.047	0.343	—
Age	0.001	0.010	0.001	0.886	—
Sexual minority	−0.010	0.057	−0.010	0.859	—
Gender minority	0.071	0.061	0.071	0.242	—
Black	0.108	0.068	0.108	0.114	—
Asian	0.028	0.067	0.028	0.670	—
American Indian	0.029	0.117	0.029	0.805	—
Latino	0.078	0.057	0.078	0.168	—
Food insecurity	−0.004	0.026	−0.004	0.878	—
Financial instability	−0.004	0.028	−0.004	0.880	—
Mental health care barriers	−0.010	0.024	−0.010	0.675	—
Poor home conditions	0.038	0.026	0.038	0.135	—
Neighborhood disorder	−0.028	0.025	−0.028	0.259	—
Discrimination	0.084	0.025	0.084	0.001	—
**Baseline mental health / adversity**					
Anger / irritability	0.274	0.025	0.274	< .001	—
Adversity	0.029	0.028	0.029	0.298	—
**Strengths**					
Suicide prevention exposure	0.107	0.076	0.107	0.159	.361
Mentor	−0.091	0.048	−0.091	0.058	.168
Activism	0.008	0.024	0.008	0.756	.860
**Self-care**	**0.070**	**0.025**	**0.070**	**0.006**	**.031**
Support receipt	0.025	0.033	0.025	0.443	.640
Support seeking	−0.071	0.040	−0.071	0.078	.212
Hope	−0.068	0.036	−0.068	0.060	.168
Purpose in life	0.027	0.035	0.027	0.440	.640
Social connectedness	0.025	0.023	0.025	0.283	.499
**Self-compassion**	**−0.152**	**0.028**	**−0.152**	**< .001**	**< .001**
Use of healthy built environment	−0.053	0.024	−0.053	0.025	.097
**Positive affect**	**−0.085**	**0.028**	**−0.085**	**0.002**	**.016**

Note. Unstandardized regression coefficients (B) and standardized coefficients (β) from the final 3-block model. Outcome standardized (z). Benjamini–Hochberg false discovery rate (FDR) correction was applied across the 120 strength-by-domain tests (12 strengths × 10 symptom domains); FDR-adjusted p values are therefore reported only for the strength coefficients. Bold indicates strength associations that remained statistically significant after FDR correction (pFDR < .05).

Model fit: Block 1: $R^2 = 0.087$, Block 2: $R^2 = 0.192$, Block 3: $R^2 = 0.244$; ΔR^2 (Block 3) = 0.052.

Supplemental Table S7

Nested Linear Regression Examining Wave 5 Mania/Hypomania Symptoms (N = 1,584)

Construct	B	SE	β	P value	FDR-adjusted P value
**Demographics / Identity / SDOH**					
Female	−0.235	0.052	−0.235	< .001	—
Age	−0.041	0.011	−0.041	< .001	—
Sexual minority	−0.093	0.060	−0.093	0.121	—
Gender minority	0.046	0.064	0.046	0.472	—
Black	0.266	0.072	0.266	< .001	—
Asian	0.200	0.070	0.200	0.004	—
American Indian	−0.316	0.124	−0.316	0.011	—
Latino	0.085	0.060	0.085	0.158	—
Food insecurity	−0.024	0.028	−0.024	0.383	—
Financial instability	0.016	0.030	0.016	0.593	—
Mental health care barriers	0.026	0.026	0.026	0.305	—
Poor home conditions	−0.021	0.027	−0.021	0.442	—
Neighborhood disorder	0.025	0.026	0.025	0.328	—
Discrimination	0.033	0.026	0.033	0.215	—
**Baseline mental health / adversity**					
Mania / hypomania	0.278	0.026	0.278	< .001	—
Adversity	0.078	0.029	0.078	0.008	—
**Strengths**					
Suicide prevention exposure	−0.099	0.080	−0.099	0.217	.427
Mentor	0.026	0.051	0.026	0.614	.768
Activism	−0.017	0.026	−0.017	0.500	.687
Self-care	0.066	0.027	0.066	0.014	.059
Support receipt	−0.034	0.035	−0.034	0.325	.545
Support seeking	0.001	0.043	0.001	0.989	.993
Hope	−0.072	0.038	−0.072	0.060	.168
Purpose in life	−0.027	0.037	−0.027	0.465	.653
Social connectedness	0.006	0.024	0.006	0.814	.897
Self-compassion	−0.050	0.029	−0.050	0.086	.229
Use of healthy built environment	0.023	0.025	0.023	0.350	.568
Positive affect	0.027	0.030	0.027	0.357	.571

Note. Unstandardized regression coefficients (B) and standardized coefficients (β) from the final 3-block model. Outcome standardized (z). Benjamini–Hochberg false discovery rate (FDR) correction was applied across the 120 strength-by-domain tests (12 strengths × 10 symptom domains); FDR-adjusted p values are therefore reported only for the strength coefficients. Bold indicates strength associations that remained statistically significant after FDR correction (pFDR < .05).

Model fit: Block 1: $R^2 = 0.067$, Block 2: $R^2 = 0.146$, Block 3: $R^2 = 0.160$; ΔR^2 (Block 3) = 0.014.

Supplemental Table S8

Nested Linear Regression Examining Wave 5 Anxiety Symptoms (N = 1,584)

Construct	B	SE	β	P value	FDR-adjusted P value
**Demographics / Identity / SDOH**					
Female	0.022	0.046	0.022	0.631	—
Age	0.020	0.009	0.020	0.031	—
Sexual minority	0.078	0.052	0.078	0.135	—
Gender minority	−0.011	0.056	−0.011	0.849	—
Black	0.061	0.063	0.061	0.331	—
Asian	0.010	0.061	0.010	0.872	—
American Indian	−0.305	0.108	−0.305	0.005	—
Latino	0.042	0.052	0.042	0.421	—
Food insecurity	0.065	0.024	0.065	0.007	—
Financial instability	−0.015	0.026	−0.015	0.553	—
Mental health care barriers	0.002	0.022	0.002	0.934	—
Poor home conditions	0.019	0.024	0.019	0.422	—
Neighborhood disorder	−0.032	0.023	−0.032	0.155	—
Discrimination	0.041	0.023	0.041	0.076	—
**Baseline mental health / adversity**					
Anxiety	0.335	0.024	0.335	< .001	—
Adversity	0.051	0.026	0.051	0.049	—
**Strengths**					
Suicide prevention exposure	0.080	0.070	0.024	0.253	.471
**Mentor**	**−0.115**	**0.044**	**−0.057**	**0.009**	**.045**
Activism	0.043	0.023	0.043	0.055	.168
**Self-care**	**0.070**	**0.024**	**0.070**	**0.003**	**.018**
Support receipt	0.024	0.030	0.024	0.435	.640
Support seeking	−0.036	0.037	−0.036	0.332	.545
**Hope**	**−0.133**	**0.034**	**−0.133**	**< .001**	**.001**
Purpose in life	0.064	0.032	0.064	0.049	.163
Social connectedness	0.045	0.021	0.045	0.035	.126
**Self-compassion**	**−0.161**	**0.026**	**−0.161**	**< .001**	**< .001**
Use of healthy built environment	−0.021	0.022	−0.021	0.328	.545
**Positive affect**	**−0.090**	**0.026**	**−0.090**	**< .001**	**.005**

Note. Unstandardized regression coefficients (B) and standardized coefficients (β) from the final 3-block model. Outcome standardized (z). Benjamini–Hochberg false discovery rate (FDR) correction was applied across the 120 strength-by-domain tests (12 strengths × 10 symptom domains); FDR-adjusted p values are therefore reported only for the strength coefficients. Bold indicates strength associations that remained statistically significant after FDR correction ($p_{FDR} < .05$).

Model fit: Block 1: $R^2 = 0.130$, Block 2: $R^2 = 0.285$, Block 3: $R^2 = 0.349$; ΔR^2 (Block 3) = 0.064.

Supplemental Table S9

Nested Linear Regression Examining Wave 5 Psychosis Symptoms (N = 1,584)

Construct	B	SE	β	P value	FDR-adjusted P value
**Demographics / Identity / SDOH**					
Female	-0.041	0.049	-0.041	0.404	—
Age	-0.023	0.010	-0.023	0.027	—
Sexual minority	-0.039	0.056	-0.039	0.482	—
Gender minority	0.074	0.060	0.074	0.218	—
Black	0.087	0.067	0.087	0.199	—
Asian	0.039	0.066	0.039	0.557	—
American Indian	0.004	0.116	0.004	0.975	—
Latino	0.043	0.056	0.043	0.445	—
Food insecurity	0.024	0.026	0.024	0.352	—
Financial instability	0.020	0.028	0.020	0.463	—
Mental health care barriers	-0.013	0.024	-0.013	0.589	—
Poor home conditions	0.002	0.025	0.002	0.945	—
Neighborhood disorder	-0.016	0.024	-0.016	0.506	—
Discrimination	0.029	0.024	0.029	0.243	—
**Baseline mental health / adversity**					
Psychosis	0.255	0.024	0.255	< .001	—
Adversity	0.047	0.027	0.047	0.089	—
**Strengths**					
Suicide prevention exposure	-0.082	0.075	-0.026	0.274	.493
Mentor	-0.013	0.047	-0.007	0.778	.872
Activism	0.030	0.024	0.032	0.214	.427
Self-care	0.018	0.025	0.019	0.468	.653
Support receipt	-0.012	0.033	-0.013	0.707	.832
Support seeking	-0.001	0.040	-0.001	0.982	.993
**Hope**	**-0.177**	**0.036**	**-0.188**	**< .001**	**< .001**
Purpose in life	0.013	0.035	0.014	0.715	.833
Social connectedness	0.044	0.023	0.047	0.056	.168
Self-compassion	-0.011	0.027	-0.011	0.695	.825
Use of healthy built environment	0.012	0.023	0.012	0.614	.768
Positive affect	0.022	0.027	0.024	0.420	.640

Note. Unstandardized regression coefficients (B) and standardized coefficients (β) from the final 3-block model. Outcome standardized (z). Benjamini–Hochberg false discovery rate (FDR) correction was applied across the 120 strength-by-domain tests (12 strengths × 10 symptom domains); FDR-adjusted p values are therefore reported only for the strength coefficients. Bold indicates strength associations that remained statistically significant after FDR correction ($p_{FDR} < .05$).

Model fit: Block 1: $R^2 = 0.049$, Block 2: $R^2 = 0.126$, Block 3: $R^2 = 0.152$; ΔR^2 (Block 3) = 0.026.

Supplemental Table S10

Nested Linear Regression Examining Wave 5 Repetitive Thoughts/Behaviors (N = 1,584)

Construct	B	SE	β	P value	FDR-adjusted P value
**Demographics / Identity / SDOH**					
Female	0.045	0.044	0.045	0.301	—
Age	0.016	0.009	0.016	0.075	—
Sexual minority	0.009	0.050	0.009	0.861	—
Gender minority	0.086	0.054	0.086	0.112	—
Black	−0.013	0.061	−0.013	0.833	—
Asian	−0.040	0.059	−0.040	0.495	—
American Indian	−0.237	0.104	−0.237	0.023	—
Latino	0.121	0.050	0.121	0.016	—
Food insecurity	0.006	0.023	0.006	0.798	—
Financial instability	−0.008	0.025	−0.008	0.755	—
Mental health care barriers	−0.006	0.022	−0.006	0.769	—
Poor home conditions	0.036	0.023	0.036	0.108	—
Neighborhood disorder	−0.043	0.022	−0.043	0.050	—
Discrimination	−0.006	0.022	−0.006	0.784	—
**Baseline mental health / adversity**					
Repetitive symptoms	0.509	0.022	0.509	< .001	—
Adversity	0.032	0.025	0.032	0.193	—
**Strengths**					
Suicide prevention exposure	0.032	0.067	0.032	0.636	.787
Mentor	−0.066	0.043	−0.066	0.122	.313
Activism	0.004	0.022	0.004	0.865	.926
Self-care	0.057	0.023	0.057	0.011	.055
Support receipt	0.001	0.029	0.001	0.979	.993
Support seeking	−0.000	0.036	−0.000	0.993	.993
**Hope**	**−0.107**	**0.032**	**−0.107**	**0.001**	**.008**
Purpose in life	0.035	0.031	0.035	0.255	.467
Social connectedness	0.011	0.021	0.011	0.608	.768
Self-compassion	−0.005	0.025	−0.005	0.831	.905
Use of healthy built environment	0.032	0.021	0.032	0.133	.319
Positive affect	−0.062	0.025	−0.062	0.013	.058

Note. Unstandardized regression coefficients (B) and standardized coefficients (β) from the final 3-block model. Outcome standardized (z). Benjamini–Hochberg false discovery rate (FDR) correction was applied across the 120 strength-by-domain tests (12 strengths × 10 symptom domains); FDR-adjusted p values are therefore reported only for the strength coefficients. Bold indicates strength associations that remained statistically significant after FDR correction ($p_{FDR} < .05$).

Model fit: Block 1: $R^2 = 0.0885$, Block 2: $R^2 = 0.3430$, Block 3: $R^2 = 0.3711$; ΔR^2 (Block 3) = 0.0281.

Supplemental Table S11

Nested Linear Regression Examining Wave 5 Substance Use Count (N = 1,584)

Construct	B	SE	β	P value	FDR-adjusted P value
**Demographics / Identity / SDOH**					
Female	-0.031	0.047	-0.031	0.505	-
Age	0.034	0.010	0.034	0.001	-
Sexual minority	0.158	0.054	0.158	0.003	-
Gender minority	0.044	0.058	0.044	0.444	-
Black	-0.056	0.065	-0.056	0.386	-
Asian	-0.097	0.063	-0.097	0.126	-
American Indian	0.093	0.112	0.093	0.406	-
Latino	0.009	0.054	0.009	0.868	-
Food insecurity	-0.014	0.025	-0.014	0.576	-
Financial instability	0.015	0.027	0.015	0.588	-
Mental health care barriers	-0.042	0.023	-0.042	0.068	-
Poor home conditions	-0.006	0.024	-0.006	0.798	-
Neighborhood disorder	0.011	0.023	0.011	0.630	-
Discrimination	0.038	0.023	0.038	0.109	-
**Baseline mental health / adversity**					
Substance use	0.481	0.023	0.481	< .001	-
Adversity	0.015	0.027	0.015	0.567	-
**Strengths**					
Suicide prevention exposure	0.053	0.072	0.053	0.466	.653
Mentor	-0.030	0.046	-0.030	0.506	.687
Activism	0.007	0.023	0.007	0.760	.860
Self-care	-0.016	0.024	-0.016	0.515	.687
Support receipt	-0.016	0.032	-0.016	0.603	.768
Support seeking	0.016	0.038	0.016	0.681	.817
Hope	-0.027	0.034	-0.027	0.442	.640
Purpose in life	-0.077	0.033	-0.077	0.020	.081
Social connectedness	0.024	0.022	0.024	0.275	.493
Self-compassion	0.050	0.026	0.050	0.060	.168
**Use of healthy built environment**	**0.104**	**0.022**	**0.104**	**< .001**	**.0001**
Positive affect	0.052	0.027	0.052	0.052	.168

Note. Unstandardized regression coefficients (B) and standardized coefficients (β) from the final 3-block model. Outcome standardized (z). Benjamini-Hochberg false discovery rate (FDR) correction was applied across the 120 strength-by-domain tests (12 strengths x 10 symptom domains); FDR-adjusted p values are therefore reported only for the strength coefficients. Bold indicates strength associations that remained statistically significant after FDR correction (pFDR < .05).

Model fit: Block 1: $R^2 = 0.0796$, Block 2: $R^2 = 0.2796$, Block 3: $R^2 = 0.2976$; ΔR^2 (Block 3) = 0.0180.

Supplemental Table S12
Nested Regression With Sex Assigned at Birth Moderation of Strengths on Global Mental Health at Wave 5

Construct	Block 1: Demographics & SDoH B (SE)	P value	Block 2: + Baseline mental health & adversity B (SE)	P value	Block 3: + Strengths B (SE)	P value	Block 4: + Interactions B (SE)	P value
**Demographics / identity / SDoH**								
Female at birth	0.116 (0.048)	.015	0.019 (0.043)	.664	-0.029 (0.043)	.503	-0.128 (0.142)	.367
Age	0.000 (0.010)	.969	0.006 (0.009)	.538	0.007 (0.009)	.454	0.008 (0.009)	.361
Sexual minority identity	0.169 (0.056)	.002	0.085 (0.050)	.088	0.043 (0.049)	.379	0.033 (0.049)	.503
Gender minority identity	0.259 (0.060)	<.001	0.140 (0.054)	.009	0.085 (0.052)	.105	0.090 (0.052)	.085
Black race	0.050 (0.068)	.459	0.065 (0.061)	.281	0.105 (0.059)	.074	0.101 (0.059)	.088
Asian race	-0.091 (0.066)	.169	-0.010 (0.059)	.870	0.008 (0.057)	.891	0.012 (0.058)	.840
American Indian race	-0.051 (0.116)	.659	-0.183 (0.104)	.079	-0.191 (0.101)	.059	-0.178 (0.102)	.080
Latino ethnicity	0.056 (0.056)	.318	0.083 (0.050)	.098	0.069 (0.049)	.155	0.062 (0.049)	.208
Food insecurity	0.084 (0.026)	.001	0.032 (0.023)	.172	0.031 (0.023)	.167	0.030 (0.023)	.180
Financial instability	0.032 (0.027)	.248	-0.011 (0.025)	.670	-0.013 (0.024)	.601	-0.013 (0.024)	.589
Mental health care barriers	0.115 (0.024)	<.001	0.025 (0.022)	.243	0.005 (0.021)	.803	0.007 (0.021)	.725
Poor home conditions	0.051 (0.025)	.044	0.033 (0.023)	.148	0.033 (0.022)	.140	0.032 (0.022)	.150
Neighborhood disorder	0.018 (0.024)	.451	-0.030 (0.022)	.170	-0.025 (0.021)	.238	-0.024 (0.021)	.262
Discrimination	0.164 (0.024)	<.001	0.036 (0.022)	.107	0.034 (0.022)	.113	0.033 (0.022)	.125
**Baseline mental health / adversity**								
Global symptoms	—	—	0.463 (0.024)	<.001	0.375 (0.025)	<.001	0.375 (0.025)	<.001
Adversity	—	—	0.078 (0.025)	.002	0.068 (0.024)	.005	0.069 (0.024)	.005
**Strengths (main effects)**								
Suicide prevention receipt	—	—	—	—	0.067 (0.065)	.309	-0.012 (0.110)	.912
Mentoring	—	—	—	—	-0.120 (0.041)	.004	-0.117 (0.071)	.101
Activism	—	—	—	—	0.016 (0.021)	.441	0.004 (0.033)	.896
Self-care	—	—	—	—	0.087 (0.022)	<.001	0.035 (0.035)	.324

Construct	Block 1: Demographics & SDoH B (SE)	P value	Block 2: + Baseline mental health & adversity B (SE)	P value	Block 3: + Strengths B (SE)	P value	Block 4: + Interactions B (SE)	P value
Received social support	—	—	—	—	-0.001 (0.029)	.978	0.036 (0.037)	.332
Sought social support	—	—	—	—	-0.010 (0.035)	.776	0.017 (0.039)	.672
Hope	—	—	—	—	-0.159 (0.031)	<.001	-0.157 (0.055)	.005
Purpose in life	—	—	—	—	0.027 (0.030)	.368	0.084 (0.053)	.110
Social connectedness	—	—	—	—	0.039 (0.020)	.052	0.024 (0.036)	.503
Self-compassion	—	—	—	—	-0.098 (0.024)	<.001	-0.067 (0.041)	.105
Healthy environment use	—	—	—	—	0.005 (0.020)	.791	0.043 (0.034)	.209
Recovering positive affect	—	—	—	—	-0.048 (0.024)	.047	-0.119 (0.043)	.006
**Strength × female-at-birth interactions**								
Suicide prevention receipt × female at birth	—	—	—	—	—	—	0.113 (0.136)	.614
Mentoring × female at birth	—	—	—	—	—	—	0.002 (0.087)	.983
Activism × female at birth	—	—	—	—	—	—	0.017 (0.041)	.757
Self-care × female at birth	—	—	—	—	—	—	0.084 (0.044)	.231
Received social support × female at birth	—	—	—	—	—	—	0.071 (0.059)	.480
Sought social support × female at birth	—	—	—	—	—	—	-0.100 (0.093)	.502
Hope × female at birth	—	—	—	—	—	—	-0.004 (0.067)	.983
Purpose in life × female at birth	—	—	—	—	—	—	-0.081 (0.064)	.466

Construct	Block 1: Demographics & SDoH B (SE)	P value	Block 2: + Baseline mental health & adversity B (SE)	P value	Block 3: + Strengths B (SE)	P value	Block 4: + Interactions B (SE)	P value
Social connectedness × female at birth	—	—	—	—	—	—	0.020 (0.043)	.752
Self-compassion × female at birth	—	—	—	—	—	—	-0.053 (0.050)	.502
Healthy environment use × female at birth	—	—	—	—	—	—	-0.055 (0.042)	.466
Recovering positive affect × female at birth	—	—	—	—	—	—	0.102 (0.052)	.231
**Model fit (nested blocks)**								
R^2	.1747		.3449		.3951		.4009	
ΔR^2	—		.1702		.0503		.0057	
P value	<.001		<.001		<.001		.256	

Note. Values are unstandardized regression coefficients (B) with standard errors (SE) in parentheses. For the interaction rows, the Block 4 P values are Benjamini-Hochberg false discovery rate (FDR)-adjusted values. Interaction coefficients represent the difference in the strength slope for participants assigned female at birth relative to those assigned male at birth; the female-at-birth slope equals the corresponding Block 4 main-effect coefficient plus the interaction coefficient. No sex-at-birth interaction remained statistically significant after FDR correction. N = 1,584.

Supplemental Table S13

Nested Regression Examining Gender Identity Moderation of Strengths on Wave 5 Global Mental Health Symptoms (N = 1,584)

Construct	Block 1: Demographics & SDoH B (SE)	P value	Block 2: + Baseline mental health & adversity B (SE)	P value	Block 3: + Strengths B (SE)	P value	Block 4: + Interactions B (SE)	P value / FDR-adjusted P value
**Demographics / identity / SDoH**								
Female at birth	0.116 (0.048)	.015	0.019 (0.043)	.664	-0.029 (0.043)	.503	-0.028 (0.043)	.516
Age	0.000 (0.010)	.969	0.006 (0.009)	.538	0.007 (0.009)	.454	0.007 (0.009)	.399
Sexual minority identity	0.169 (0.056)	.002	0.085 (0.050)	.088	0.043 (0.049)	.379	0.040 (0.049)	.416
Gender minority identity	0.259 (0.060)	< .001	0.140 (0.054)	.009	0.085 (0.052)	.105	0.131 (0.162)	.418
Black race	0.050 (0.068)	.459	0.065 (0.061)	.281	0.105 (0.059)	.074	0.111 (0.059)	.057
Asian race	-0.091 (0.066)	.169	-0.010 (0.059)	.870	0.008 (0.057)	.891	0.023 (0.057)	.685
American Indian race	-0.051 (0.116)	.659	-0.183 (0.104)	.079	-0.191 (0.101)	.059	-0.224 (0.102)	.028
Latino ethnicity	0.056 (0.056)	.318	0.083 (0.050)	.098	0.069 (0.049)	.155	0.069 (0.049)	.159
Food insecurity	0.084 (0.026)	.001	0.032 (0.023)	.172	0.031 (0.023)	.167	0.030 (0.023)	.188
Financial instability	0.032 (0.027)	.248	-0.011 (0.025)	.670	-0.013 (0.024)	.601	-0.014 (0.024)	.559
Mental health care barriers	0.115 (0.024)	< .001	0.025 (0.022)	.243	0.005 (0.021)	.803	0.006 (0.021)	.790
Poor home conditions	0.051 (0.025)	.044	0.033 (0.023)	.148	0.033 (0.022)	.140	0.033 (0.022)	.130
Neighborhood disorder	0.018 (0.024)	.451	-0.030 (0.022)	.170	-0.025 (0.021)	.238	-0.025 (0.021)	.236
Discrimination exposure	0.164 (0.024)	< .001	0.036 (0.022)	.107	0.034 (0.022)	.113	0.033 (0.022)	.133
**Baseline mental health / adversity**								
Global symptoms	—	—	0.463 (0.024)	< .001	0.375 (0.025)	< .001	0.380 (0.025)	< .001
Adversity	—	—	0.078 (0.025)	.002	0.068 (0.024)	.005	0.062 (0.024)	.011
**Strengths (main effects)**								
Suicide prevention receipt	—	—	—	—	0.067 (0.065)	.309	0.093 (0.073)	.207
Mentoring	—	—	—	—	-0.120 (0.041)	.004	-0.155 (0.051)	.002
Activism	—	—	—	—	0.016 (0.021)	.441	-0.020 (0.026)	.448
Self-care	—	—	—	—	0.087 (0.022)	< .001	0.111 (0.026)	< .001
Received social support	—	—	—	—	-0.001 (0.029)	.978	0.027 (0.037)	.459
Sought social support	—	—	—	—	-0.010 (0.035)	.776	-0.035 (0.038)	.350
Hope	—	—	—	—	-0.159 (0.031)	< .001	-0.144 (0.039)	< .001
Purpose in life	—	—	—	—	0.027 (0.030)	.368	-0.026 (0.037)	.480
Social connectedness	—	—	—	—	0.039 (0.020)	.052	0.025 (0.025)	.301
Self-compassion	—	—	—	—	-0.098 (0.024)	< .001	-0.119 (0.028)	< .001

Construct	Block 1: Demographics & SDoH B (SE)	P value	Block 2: + Baseline mental health & adversity B (SE)	P value	Block 3: + Strengths B (SE)	P value	Block 4: + Interactions B (SE)	P value / FDR-adjusted P value
Healthy environment use	—	—	—	—	0.005 (0.020)	.791	0.029 (0.024)	.214
Recovering positive affect	—	—	—	—	-0.048 (0.024)	.047	-0.006 (0.029)	.821
**Strength × gender minority identity interactions**								
Suicide prevention receipt × GM	—	—	—	—	—	—	-0.099 (0.157)	.720
Mentoring × GM	—	—	—	—	—	—	0.110 (0.086)	.466
Activism × GM	—	—	—	—	—	—	0.100 (0.043)	.142
Self-care × GM	—	—	—	—	—	—	-0.071 (0.047)	.425
Received social support × GM	—	—	—	—	—	—	-0.057 (0.058)	.514
Sought social support × GM	—	—	—	—	—	—	0.192 (0.121)	.407
Hope × GM	—	—	—	—	—	—	-0.040 (0.065)	.720
Purpose in life × GM	—	—	—	—	—	—	0.161 (0.064)	.104
Social connectedness × GM	—	—	—	—	—	—	0.033 (0.041)	.621
Self-compassion × GM	—	—	—	—	—	—	0.073 (0.053)	.466
Healthy environment use × GM	—	—	—	—	—	—	-0.099 (0.045)	.169
Recovering positive affect × GM	—	—	—	—	—	—	-0.136 (0.053)	.104
**Model fit (nested blocks)**								
R^2	.1747		.3449		.3951		.4078	
ΔR^2	—		.1702		.0503		.0126	
P value	< .001		< .001		< .001		.0011	

Note. Values are unstandardized regression coefficients (*B*) with standard errors (SE) in parentheses. For interaction terms, coefficients represent the difference in the association between each strength and Wave 5 global symptom severity for gender-minority participants relative to non-gender-minority participants, the reference group. *P* values for interaction terms were adjusted using the Benjamini–Hochberg false discovery rate (FDR) procedure across the 36 strength × moderator interaction tests (12 strengths × 3 moderators). No individual strength × gender minority identity interaction remained statistically significant after FDR correction.

Supplemental Table S14

Nested Regression With Sexual Identity Moderation of Strength Associations With Global Mental Health at Wave 5

Construct	Block 1 B (SE)	P value	Block 2 B (SE)	P value	Block 3 B (SE)	P value	Block 4 B (SE)	P value / FDR-adjusted P
**Demographics / identity / SDoH**								
Female at birth	0.116 (0.048)	.015	0.019 (0.043)	.664	−0.029 (0.043)	.503	−0.017 (0.043)	.698
Age	0.000 (0.010)	.969	0.006 (0.009)	.538	0.007 (0.009)	.454	0.006 (0.009)	.509
Sexual minority identity (moderator)	0.169 (0.056)	.002	0.085 (0.050)	.088	0.043 (0.049)	.379	0.102 (0.136)	.453
Gender minority identity	0.259 (0.060)	< .001	0.140 (0.054)	.009	0.085 (0.052)	.105	0.070 (0.053)	.187
Black race	0.050 (0.068)	.459	0.065 (0.061)	.281	0.105 (0.059)	.074	0.110 (0.058)	.061
Asian race	−0.091 (0.066)	.169	−0.010 (0.059)	.870	0.008 (0.057)	.891	0.022 (0.057)	.694
American Indian race	−0.051 (0.116)	.659	−0.183 (0.104)	.079	−0.191 (0.101)	.059	−0.227 (0.101)	.025
Latino ethnicity	0.056 (0.056)	.318	0.083 (0.050)	.098	0.069 (0.049)	.155	0.077 (0.049)	.115
Food insecurity	0.084 (0.026)	.001	0.032 (0.023)	.172	0.031 (0.023)	.167	0.031 (0.022)	.162
Financial instability	0.032 (0.027)	.248	−0.011 (0.025)	.670	−0.013 (0.024)	.601	−0.013 (0.024)	.597
Mental health care barriers	0.115 (0.024)	< .001	0.025 (0.022)	.243	0.005 (0.021)	.803	0.005 (0.021)	.815
Poor home conditions	0.051 (0.025)	.044	0.033 (0.023)	.148	0.033 (0.022)	.140	0.029 (0.022)	.185
Neighborhood disorder	0.018 (0.024)	.451	−0.030 (0.022)	.170	−0.025 (0.021)	.238	−0.021 (0.021)	.317
Discrimination	0.164 (0.024)	< .001	0.036 (0.022)	.107	0.034 (0.022)	.113	0.032 (0.022)	.142
**Baseline mental health / adversity**								
Baseline global symptoms	—	—	0.463 (0.024)	< .001	0.375 (0.025)	< .001	0.381 (0.025)	< .001
Baseline adversity	—	—	0.078 (0.025)	.002	0.068 (0.024)	.005	0.061 (0.024)	.012
**Strengths (main effects)**								
Suicide prevention receipt	—	—	—	—	0.067 (0.065)	.309	0.092 (0.089)	.302
Mentoring	—	—	—	—	−0.120 (0.041)	.004	−0.145 (0.063)	.022
Activism	—	—	—	—	0.016 (0.021)	.441	−0.071 (0.031)	.023
Self-care	—	—	—	—	0.087 (0.022)	< .001	0.100 (0.031)	.001
Received social support	—	—	—	—	−0.001 (0.029)	.978	0.046 (0.047)	.323
Sought social support	—	—	—	—	−0.010 (0.035)	.776	−0.050 (0.041)	.220
Hope	—	—	—	—	−0.159 (0.031)	< .001	−0.147 (0.049)	.002
Purpose in life	—	—	—	—	0.027 (0.030)	.368	−0.016 (0.046)	.728
Social connectedness	—	—	—	—	0.039 (0.020)	.052	0.038 (0.030)	.202
Self-compassion	—	—	—	—	−0.098 (0.024)	< .001	−0.124 (0.034)	< .001
Healthy environment use	—	—	—	—	0.005 (0.020)	.791	0.020 (0.028)	.466
Recovering positive affect	—	—	—	—	−0.048 (0.024)	.047	0.045 (0.035)	.208
**Strength × sexual minority interactions**								
Suicide prevention receipt × sexual minority	—	—	—	—	—	—	−0.067 (0.130)	.752
Mentoring × sexual minority	—	—	—	—	—	—	0.039 (0.083)	.752
**Activism × sexual minority**	**—**	**—**	**—**	**—**	**—**	**—**	**0.157 (0.041)**	**.006**

Construct	Block 1<br>B (SE)	P value	Block 2<br>B (SE)	P value	Block 3<br>B (SE)	P value	Block 4<br>B (SE)	P value / FDR-adjusted P
Self-care × sexual minority	—	—	—	—	—	—	−0.019 (0.042)	.752
Received social support × sexual minority	—	—	—	—	—	—	−0.067 (0.059)	.502
Sought social support × sexual minority	—	—	—	—	—	—	0.199 (0.102)	.231
Hope × sexual minority	—	—	—	—	—	—	−0.022 (0.063)	.794
Purpose in life × sexual minority	—	—	—	—	—	—	0.078 (0.061)	.466
Social connectedness × sexual minority	—	—	—	—	—	—	−0.002 (0.040)	.983
Self-compassion × sexual minority	—	—	—	—	—	—	0.047 (0.048)	.514
Healthy environment use × sexual minority	—	—	—	—	—	—	−0.043 (0.040)	.502
**Recovering positive affect × sexual minority**	**—**	**—**	**—**	**—**	**—**	**—**	**−0.168 (0.048)**	**.008**
**Model fit**								
R^2	.1747	—	.3449	—	.3951	—	.4090	—
ΔR^2	—	—	.1702	—	.0503	—	.0138	—
P value	< .001	—	< .001	—	< .001	—	.0003	—

Note. Values are unstandardized regression coefficients (B) with standard errors (SE) in parentheses. Interaction rows reflect differences for sexual minority relative to heterosexual youth. P values for the 36 interaction terms across the three moderation analyses were adjusted using the Benjamini–Hochberg false discovery rate (FDR) procedure. In the interaction section, the P-value column reports FDR-adjusted P values. Bold indicates interactions that remained statistically significant after FDR correction.
